# School-based randomized controlled trials for ADHD and accompanying impairments: a systematic review and meta-analysis

**DOI:** 10.3389/fpsyg.2025.1611145

**Published:** 2025-07-21

**Authors:** Beliz Yegencik, Beth T. Bell, Emre Deniz

**Affiliations:** Department of Education, The University of York, York, United Kingdom

**Keywords:** attention deficit hyperactivity disorder, school, intervention, systematic review, meta-analysis

## Abstract

**Introduction:**

Children and young people with Attention Deficit Hyperactivity Disorder (ADHD) face disproportionate challenges due to core symptoms of ADHD (i.e., inattention and hyperactivity) interfering with academic, social, and behavioural functioning. The significant rise in the prevalence of ADHD and difficulties experienced by children and young people over recent years has put a lot more emphasis on school-based interventions. Despite this, and the tremendous amount of randomised controlled trials (RCTs) reporting school-based interventions for ADHD over the past decades, there has been no systematic reporting of the pooled effects of such trials in the literature.

**Methods:**

This study seeks, for the first time, to report the effects of school-based RCTs on the core symptoms of ADHD and other difficulties, i.e., academic, social, emotional and behavioural (i.e., externalising). Search was performed in three journals, for interventions from 1980 to 2024 that targeted school-aged children (4 to 18 years old) with ADHD.

**Results:**

In total, 26 randomised controlled trials met the inclusion criteria, 22 (n = 1962) of which were included in our meta-analyses. Our findings showed that school-based based randomised controlled trials were effective in improving combined ADHD (*d* = −0.28, *p* < 0.0001), inattention (*d* = −0.33, *p* < 0.0001), academic performance (*d* = 0.37, *p* < 0.0001), and social skills (*d* = 0.28, *p* < 0.001), and reducing externalising problems (*d* = −0.32, *p* = 0.001). There was no significant effect for hyperactivity/impulsivity (*d* = −0.09, *p* = 0.22)

**Discussion:**

Our findings showed that school-based randomised controlled trials improve a range of difficulties experienced by children and young people with ADHD, but lack focus on hyperactivity/impulsivity. Future interventions would benefit from a more comprehensive focus on hyperactivity/impulsivity for system-wide improvement. Issues related to high levels of heterogeneity and potential reporter bias are further discussed.

## Introduction

Attention Deficit and Hyperactivity Disorder (ADHD) is one of the most prevalent neurodevelopmental conditions, with latest figures showing a global rate of 8% in children and adolescents (Ayano et al., 2023). It is characterised by two core difficulties: inattentiveness, which are difficulties with sustaining attention, organisational skills, and following instructions; and hyperactivity/impulsivity, which presents as acting before thinking, excessive talking, and fidgeting (National Institute of Mental Health, 2024). Among the presentation types of ADHD, combined inattention and hyperactivity is the most prevalent (Ayano et al., 2020; Wilens et al., 2009). Most children and young people (CYP) with ADHD have at least one of the comorbid behavioural (e.g., conduct problems), emotional (e.g., anxiety, depression), or neurodevelopmental (e.g., autism) conditions (Centers for Disease Control and Prevention, 2024; Gillberg et al., 2004). Such difficulties, alongside core symptoms of ADHD, often present additional negative consequences in other areas of functioning, such as social and academic skills (Mak et al., 2022; Wehmeier et al., 2010; Jensen et al., 2001).

In terms of social difficulties, CYP with ADHD experience higher levels of peer rejection and generally have less friends than their neurotypical peers (Ferretti et al., 2019; Singh et al., 2015; Mrug et al., 2012). This may be due to several reasons, such as difficulties in taking turns in games or in conversations, or in making and maintaining friendships; disruptive and impulsive behaviours, and having poor emotion regulation and self-esteem (Faraone et al., 2021; Ferretti et al., 2019; Van Stralen, 2016). Children with ADHD also tend to struggle with their interactions and relationships with adults. They appear to have more conflict with their parents and perceive less social support from their teachers (Faraone et al., 2021; Ferretti et al., 2019; Singh et al., 2015; Mrug et al., 2012). Such social experiences may be worsened due to co-occurring difficulties, such as autism spectrum conditions.

Children with ADHD also struggle academically, usually due to the core symptoms of ADHD interfering with skills that require sustained attention and behavioural control, such as executive function skills, inhibition and attentional control, and working memory (Fan and Wang, 2022; Roselló et al., 2020; Schreiber et al., 2014; Miller et al., 2011; Brocki et al., 2010). These are essential for academic tasks that require high attentional skills, such as maths and reading, also in keeping attendance and managing homework (Faraone et al., 2021; Loe and Feldman, 2007). Hence, CYP often perform below average in academic attainment and are at high risk for school dropout (Arnold et al., 2020; Fredriksen et al., 2014), which can lead to difficulties with occupational performance, job stability, and career development (Gordon and Fabiano, 2019). Taken together, compared to their non-ADHD peers, children and adolescents with ADHD are at a greater risk for poor academic outcomes and future achievement.

ADHD and accompanying difficulties often become more evident once children reach school age (Salari et al., 2023) even though the symptoms can emerge earlier (Miller et al., 2023). One reason for this is because, during preschool years, children are not expected to deal with complex daily tasks and social interactions as they are when they start school. Therefore, being presented with additional requirements in educational settings tends to reveal the difficulties associated with ADHD. Another reason is that teachers might be better equipped to identify children that significantly differ from their non-ADHD peers than parents (Nafi et al., 2020) and in general, teachers play a key role in recognising children’s difficulties (Akdağ, 2023; Daley and Birchwood, 2010). Given that core ADHD symptoms tend to unfold within school settings where children spend a substantial amount of time, schools are often expected to play a central role in supporting these children and adolescents.

A wide range of school-based interventions has been developed and implemented to support various areas of functioning in CYP with ADHD (Daley and Birchwood, 2010; DuPaul et al., 2012; DuPaul and Eckert, 1997), one of which is multimodal treatments with a direct focus on the core difficulties of ADHD. Multimodal approaches also target other areas of functioning, such as cognitive skills (Capodieci et al., 2017; Egeland et al., 2013), auditory and visual attention (Chacona, 2008), organisation and planning (Langberg et al., 2012), academic (Owens et al., 2020) and social skills (Evans et al., 2023; Lan et al., 2018), which would indirectly promote improvements in the core difficulties of ADHD. These interventions often include behavioural components, such as antecedent and consequent-based strategies, which manipulate the environment to reduce or encourage a certain behaviour (DuPaul et al., 2011). There are also some interventions that aim to create a bridge between school and home environment (e.g., Pfiffner et al., 2016), some of which include components like psychoeducation for parents and teachers, where they are taught behavioural modification techniques (Hornstra et al., 2023). Similarly, interventions focusing on cognitive training (e.g., neurofeedback, executive functions), therapeutic approaches (e.g., mindfulness therapies), and educational programs in schools (e.g., individualised programs, accommodations) are also common (Nazarova et al., 2022; Dahl et al., 2020; Ferrin et al., 2016; Fabiano et al., 2009).

School-based ADHD interventions have many advantages, such as accessibility, cost, and feasibility as they can be embedded into daily classroom activities and delivered at a large scale. Classroom-based psychosocial interventions are found to be beneficial for improving ADHD symptoms, with effects transcending to conduct problems, social skills, and on-task behaviour (Fabiano et al., 2015; Daley et al., 2014; DuPaul et al., 2012; Miranda et al., 2006). They are promising to improve certain outcomes especially where clinical interventions (e.g., medication use) have very small effects, such as academic performance (Fabiano et al., 2009). However, research shows that the scope and mechanisms of interventions for ADHD vary significantly, and more research is needed to understand if and how these interventions work to tackle difficulties experienced by children with ADHD.

### Rationales for the current study

There have been many attempts to systematically investigate the effectiveness of school-based interventions on core ADHD symptoms and accompanying impairments. While the earliest form of such meta-analyses showed that school-based interventions are somewhat beneficial for improving core symptoms of ADHD and academic skills (DuPaul and Eckert, 1997), later evidence with control conditions found no significant effects of such interventions (DuPaul et al., 2012). A more up-to-date evaluation (Moore et al., 2018) has also shown inconsistent findings for the effectiveness of school-based ADHD interventions, alongside reporting rater bias. While these reviews have provided insights into the effectiveness of such interventions for ADHD, several important gaps remain. For instance, Moore et al. (2018) reported the efficacy of randomised trials by intervention type which considerably reduced the sample power and lacked a general picture of overall effectiveness of such interventions. Additionally, DuPaul et al. (2012) had methodological limitations such as reporting trials with various designs, including those with single-subject. Hence, despite an abundance of research trials delivering school-based interventions to improve the core difficulties of ADHD and accompanying impairments, the overall efficacy of such trials has remained unclear as past evidence appears inconclusive.

To close this gap in the literature and provide up-to-date evidence, we conducted the most comprehensive review to date, investigating the efficacy of school-based randomised controlled trials (RCTs) targeting core difficulties of ADHD and accompanying impairments published between 1980 and 2024. In doing this, we sought to address the following research questions: (1) What are the key characteristics of school-based randomised controlled trials targeting core difficulties of ADHD and accompanying impairments? (2) How effective, if at all, are school-based randomised controlled trials in improving core difficulties of ADHD and accompanying impairments? (3) Does the type of reporter (e.g., teacher, parent) moderate the pooled effect size of the school-based interventions for ADHD and accompanying impairments? (4) Does the efficacy of school-based interventions change depending on school age (i.e., primary vs. secondary)? By combining data from high quality RCTs, this study seeks a more cumulative approach in answering whether school-based interventions work for children and young people with ADHD and aims to inform school practices, policy decisions, and future interventions.

## Methods

### Target design

This review targeted randomised controlled trials. Hence, studies with all other experimental designs (e.g., quasi-experimental, within/between-subject, pre- and post-test) were kept out of the scope of the current study.

### Target sample

The target sample was school-aged children (4–18 years old; age of compulsory education in the UK), who had a formal diagnosis of ADHD or those who met diagnostic cut-off scores on a standardised ADHD scale.

### Outcome variables

The target primary outcomes of the current review were core ADHD difficulties (i.e., inattention, hyperactivity/impulsivity, and combined ADHD). The secondary target outcomes were behavioural difficulties accompanying ADHD, i.e., difficulties with academic and social skills and externalising problems. In this review, externalising problems are conceptualised as the symptoms of Oppositional Defiant Disorder (ODD) and Conduct Disorder (CD). Studies that did not report any of the primary outcomes were excluded from the current review.

### Intervention characteristics

The target interventions were those that took place in school settings and targeted ADHD and accompanying difficulties of school-aged children with ADHD. Interventions that were outside school settings (e.g., home) and those that were focused on other target groups (e.g., parents) were excluded, unless they had a child component at school and the child outcomes were reported separately (e.g., interventions with a component of parental training).

### Study selection procedure

This review followed the Preferred Reporting Items for Systematic Reviews and Meta-Analyses (PRISMA, Shamseer et al., 2015) guideline. Following the PRISMA principles, the first author BY screened the ERIC, PsycINFO, and PubMed databases using Boolean search strings. To increase the likelihood of the identification of the relevant studies, following search strings were defined based on the target sample and intervention: “adhd” AND “school” AND “intervention.” The following synonymous terms were also searched for maximising the study identification results: For “school”; school-based OR classroom OR classroom-based; for “ADHD”; attention deficit*, OR hyperactiv*; and for “intervention”; treatment OR education OR training (see Table 1). Finally, all search strings were screened in all fields (i.e., title, abstract, and full text). All study titles and abstracts were screened and organised systematically in Endnote. If needed, any discrepancies or uncertainties regarding the eligibility of a paper were resolved through a discussion between the first and third author. To be included, a study had to meet the following criteria: (a) reported a randomised controlled trial, (b) delivered a child-focused intervention in a school setting, (c) consisted of children clinically diagnosed with ADHD or used a valid ADHD assessment tool, (d) reported core ADHD difficulties (i.e., inattention, hyperactivity/impulsivity, ADHD), and (e) published in English between 1980 and 2024.

**Table 1:** Study selection key term strings.

Neurodiversity		Context		Intervention
attention deficit and hyperactivity disorder” OR “adhd” OR “attention deficit*“OR hyperactiv*	AND	“school” OR “school-based” OR “classroom” OR “classroom-based”	AND	“intervention*” OR “treatment” OR “treat*” OR “training” OR “train*” OR “education*”

### Data extraction

We extracted two types of data, descriptive and quantified. In terms of descriptive data, we extracted information related to the following: sample characteristics (including gender distribution and medication use), experimental design, intervention type, intervention duration, intervention setting/school type, intervention administrator, outcome reporter, and intervention components. In terms of quantified data, we extracted sample sizes, means and standard deviations, and, where necessary, standard errors and reported effect sizes. Descriptive information and quantified data for studies that were published pre-2018 were obtained from the most up-to-date systematic review and meta-analysis (i.e., Moore et al., 2018). For post-2018 studies, the first author extracted relevant descriptive information and quantified data for each target outcome. In case there were multiple reported outcomes per domain that were reported by different reporters (e.g., parent and teacher reports on academic skills), we extracted all outcomes and conducted the meta-analyses with first reported outcomes per rater in each domain. However, in cases where there were multiple reported outcomes per domain by the same reporter (e.g., parental reports on two academic scales), we only extracted the first reported outcome to prevent selective outcome reporting; an approach that has been consistently applied in previous studies (i.e., Francis et al., 2022; Deniz et al., 2024).

### Quality appraisal

The methodological quality of the included studies, published post-2018, was evaluated by the first author. For this we used the adapted Cochrane risk of bias tool (Higgins et al., 2011). The answers included Yes, No, Not applicable, and No response in the following categories: randomisation and allocation concealment (selection bias), blinding (detection bias), response rate and fidelity (attrition bias), follow up results, missing outcome data and selective reporting. For pre-2018 studies, we extracted quality appraisals from Moore et al.’s paper (2018).

### Data analysis

All analyses were performed in R (Version 4.2.1). Due to high heterogeneity, which is a common issue in the field (Deniz et al., 2024), a random effect model with inverse-variance weighted was utilised. Furthermore, standardised mean differences were reported using Cohen’s *d* and heterogeneity was reported using I^2^ statistics. Scales for ADHD and externalising problems were coded in the same direction with lower scores indicating lower levels of difficulties. That is, a negative effect size indicates reduced level of problems for ADHD and externalising problems. For academic and social skills, a positive score means improvement in the treatment group. Regarding the outcome type, some studies reported core difficulties of ADHD separately, that is, inattention, hyperactivity/impulsivity. In cases where a study did not report an overall ADHD outcome, we combined inattention and hyperactivity subscales and derived combined ADHD scores to boost the sample power. Additionally, we performed meta-regression analyses to report whether the efficacies of trials were moderated by the type of reporter (i.e., child, parent, teacher, and observer) or school type (i.e., primary school: 4–11 years old, secondary school: 12–17 years old). Finally, publication bias was reported by visually inspecting funnel plot asymmetry and using Egger’s test.

## Results

### Study selection results

**Figure 1:**
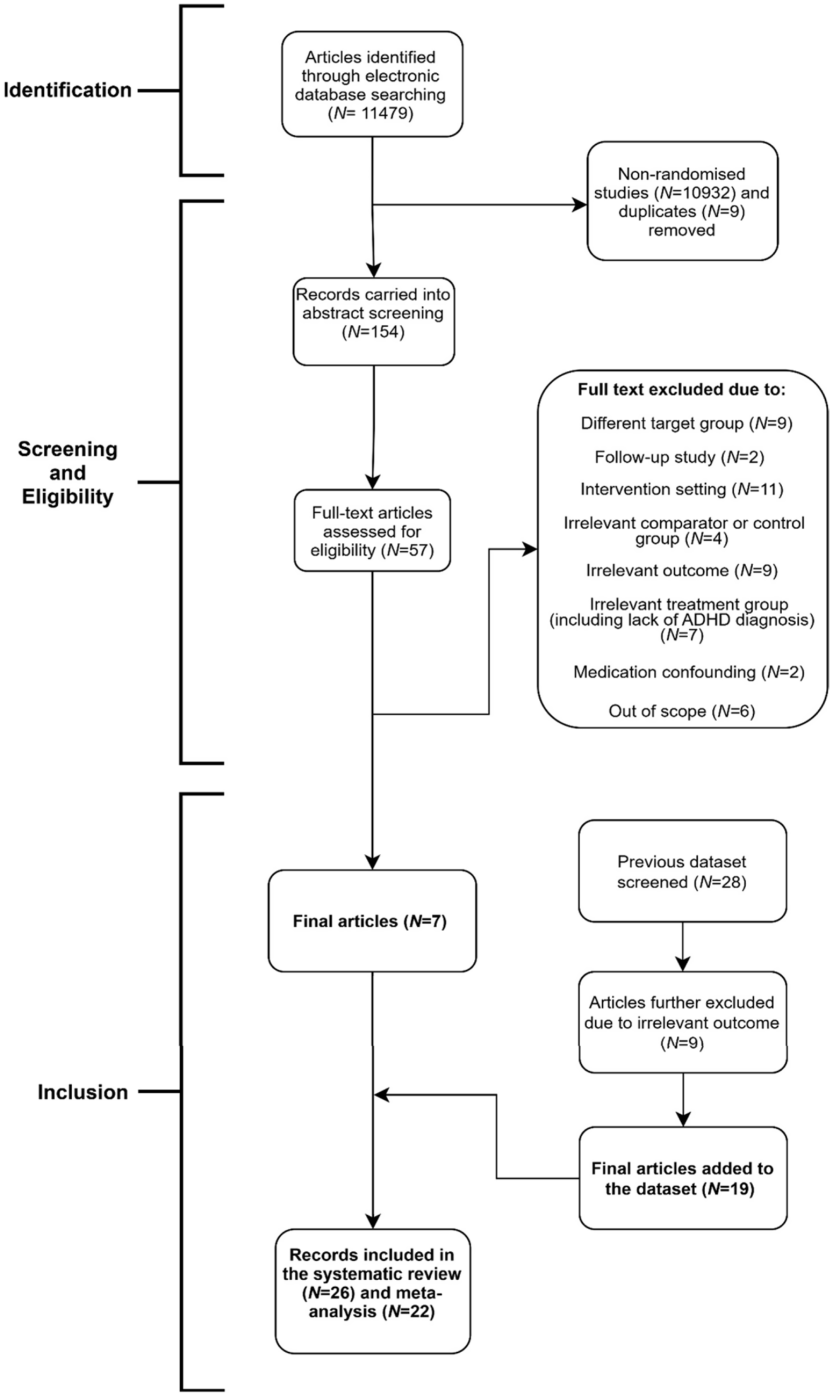
Prisma flow diagram.

Boolean search strings identified 11479 studies since 2018 in the ERIC, PsycINFO, and PubMed databases. As the first step, a filter on PubMed, along with a title and abstract screening on ERIC and PsycINFO to eliminate non-randomised studies were applied, which resulted in the exclusion of 10932 studies. Duplicates (*k* = *9*) were also removed. Hence, 538 studies were title screened. Of these, 154 studies were carried into abstract screening, 97 of which were further excluded due to irrelevance. Therefore, remaining 57 studies were full-text screened which resulted in the exclusion of 50 studies due to trials not meeting the inclusion criteria for the following reasons: intervention setting (*k* = 11), sample characteristics (*k* = 7), study aims (*k* = 6), study design (*k* = 4), target group (*k* = 9), target outcome (*k* = 9), and confounding factors (*k* = 2). Finally, two studies reported follow-up results of their precedents, hence, were excluded due to lack of follow up analysis in this paper. Additionally, one study (Capodieci et al., 2017) was excluded due to significant baseline inequivalence between the intervention and control groups but was kept for systematic review. Finally, 28 studies that were included in Moore et al. (2018) were further screened against our eligibility criteria, nine of which were excluded, while three of these were only included in the systematic review, due to inadequate information on interventions (*k* = 1), pre-test differences between groups (*k* = 1) (Seeley et al., 2009), and small sample size (*k* = 1). Hence, 26 studies (pre-2018 = 19, post-2018 = 7) were included in this systematic review, 22 of which were carried into the meta-analysis. Detailed information on database screening can be seen in the PRISMA flow diagram (see Figure 1).

### Study characteristics

The characteristics of the included studies are summarised here in brief. Detailed information is shown in Table 2. The included studies (*k* = 26) had a total sample of 2102 participants. The majority of studies (*N* = 24) were journal articles, while two studies were parts of dissertations, of which 18 were conducted in the USA, and the rest were from Iran (*k* = 2), China (*k* = 1), Italy (*k* = 1), Norway (*k* = 1), USA & Canada (*k* = 1), USA & China (*k* = 1), and USA and Mexico (*k* = 1). Finally, most interventions (*k* = 19) were conducted in primary schools, highlighting their focus on early intervention strategies.

**Table 2:** Intervention demographics of included studies.

Study details	Country	Type of control	Sample size	School level	Percentage of female participants	Percentage on medication for ADHD	ADHD subtype
Bloomquist et al. (1991)	USA	WLC	24	Primary school	31%	0%	NR
Capodieci et al. (2017)	Italy	CC	34	Primary school	18%	0%	NR
Chacona (2008)	USA	TAU	60	Primary school	30%	NR	100% inattentive
Cloward (2003)	USA	PLCB	8	Primary school	38%	NR	NR
Egeland et al. (2013)	Norway	TAU	75	Primary school	27%	69%	100% combined
Evans et al. (2011)	USA	CC	49	Secondary school	29%	31%	NR
Evans et al. (2014)	USA	CC	36	Secondary school	17%	50%	81% inattentive, 19% combined
Evans et al. (2016)	USA	CC	326	Secondary school	29%	47%	51% inattentive, 49% combined
Evans et al. (2023)	USA	CC	170	Secondary school	21%	36%	%50 combined
Fabiano et al. (2010)	USA	TAU	63	Primary school	14%	52%	87% combined, 11% inattentive, 2% hyperactive
Haack et al. (2021)	Mexico and USA	TAU	58	Primary school	28%	30%	%91 combined, 6% hyperactive
Jurbergs et al. (2010)	USA	TAU	56	Primary school	26% (of 43)	23% (of 43)	NR
Khilnani et al. (2003)	USA	WLC	30	Secondary school	20%	NR	NR
Lan et al. (2018)	China	WLC	81	Primary school	36%	NR	100% Combined
Langberg et al. (2012)	USA	WLC	47	Secondary school	23%	66%	NR
Looyeh et al. (2012)	Iran	WLC	14	Primary school	100%	0%	NR
Mikami et al. (2021)	Canada and USA	CC	134	Primary school	17%	NR	NR
Nejati et al. (2023)	Iran	CC	30	Primary school	0%	0%	NR
Omizo and Michael (1982)	USA	PLCB	32	Primary school	0%	NR	100% hyperactive
Pfiffner et al. (2016)	USA	TAU	135	Primary school	24%	8%	58% combined, 39% inattentive, 3% hyperactive
Rivera and Omizo (1980)	USA	PLCB	36	Primary school	0%	0%	100% hyperactive
Seeley et al. (2009)	USA	TAU	42	Primary school	7%	10%	52% combined, 24% inattentive, 24% hyperactive/impulsive
Sibley et al. (2018)	USA	CC	325	Secondary school	26%	42%	NR
Smith et al. (2017)	USA and China	TAU	92	Primary school	34%	16%	25% Inattentive, 15% Hyperactive, 39% Combined (of 80)
Steiner et al. (2011)	USA	WLC	41	Primary school	48%	60%	NR
Steiner et al. (2014)	USA	CC	104	Primary school	33%	49%	NR

TAU = treatment as usual; WLC = waitlist control PLCB = Placebo; CC = Community control; NR = Not reported.

### Intervention characteristics

Below information is further included in Table 3. Of 26 included studies, 21 reported intervention durations, of which, students received 38.87 hours of treatment on average. However, the total intervention length drastically varied, where the shortest intervention was 25 minutes, and the longest was 360 hours. There were six studies that conducted more than one form of intervention, and the type of interventions was also significantly different, which were: Multimodal (*k* = 11), Cognitive training (*k* = 6), Neurofeedback (*k* = 4), Self-monitoring (*k* = 2), Study and Organisational skills (*k* = 2), Daily report card (*k* = 2), Relaxation Training (*k* = 1), Social Skills Training (*k* = 1), and Task modification (*k* = 1) (Table 3). It is important to highlight that post-2018 studies delivered interventions that were not captured by pre-2018 studies (e.g., CLS- FUERTE in Mexico). In terms of who delivered the interventions, 24 out of 26 studies reported this information, where the majority (*k* = 17) were delivered by school staff, e.g., teachers and school mental health practitioners, while the rest was delivered by others, such as doctoral students (*k* = 4), and trained coaches, therapists or researchers (*k* = 3). Finally, in terms of control conditions, the most common control type was community care (*k* = 9), followed by treatment as usual (*k* = 8), waitlist control (*k* = 6), and placebo (*k* = 3).

**Table 3:** Intervention characteristics of included studies.

Study details	Intervention category	Intervention name	Involves home or parents	During school hours	Delivered by	Total hours of treatment	Intervention components
Bloomquist et al. (1991)	Self- monitoring	Multicomponent Cognitive Behavioural Training (CBT)	Yes	Yes	SMHP, teacher, student	20	Groups of 6–8 ADHD children, weekly meetings. A step-by-step guide to support problem-solving, applying these to the chosen areas (e.g., interpersonal problem-solving, anger management) through the implementation of behavioural strategies, such as role-playing, response cost and modelling. Homework assignments to practice skills.
Capodieci et al. (2017)	Cognitive training	Working memory training	No	Yes	Class teachers supervised by psychologists	16	Training in 4 blocks: 1. Introducing behavioural strategies for retaining and controlling information in working memory (WM). 2. Trainer-selected working memory exercises. Games with pencil and paper or a motor activity. 3. Six sessions for the selected WM with an interpolated task. 4. Focus on the ability to update information in WM. Each session followed a structured format: metacognitive introduction, cognitive demand explanation, practice, group work, strategic reflection, and introspective feedback. Activities aimed to enhance children’s self-awareness and strategic use of working memory in daily challenges.
Chacona (2008)	Task modification	World music drumming curriculum	No	Yes	Teacher	7	Intervention uses call and response, using “stop, look, and listen” technique. Students stop their activity to focus on the leader’s drum beats, following which they echo. Aimed at improving attention and reducing impulsivity by training focused listening and controlled behavioural responses.
Cloward (2003)	Self- monitoring	Self-monitoring	No	Yes	Teacher	NR	Two self-monitoring treatment groups responded to random beeps (10–80s apart) by marking whether they were on-task. Students were trained in task awareness, self-recording accuracy, and received teacher feedback. Practice included group instruction, daily tape use during independent work, and role-play to reinforce behavioural accuracy over two consecutive days.
Egeland et al. (2013)	Cognitive training	Cogmed’s RoboMemo program	No	Yes	Teacher	18.75	WM training used over 5–7 weeks, with daily 30–45 min sessions of 13 adaptive tasks targeting WM capacity. Tasks included digit/letter spans and visuospatial recall (static/dynamic, forward/reverse). Students received visual/auditory feedback, daily and individualised rewards, and adult supervision. Task difficulty adjusted to performance, with exercises rotated every 5 days.
Evans et al. (2011)	Combined	Challenging horizons after- school programme	Yes	No	Student	86	Held twice weekly, featuring education and interpersonal skills groups, recreation, and individual sessions. Interventions focused on improving disorganisation, study habits, and social behaviour. Students were taught academic skills (e.g., study strategies, note-taking) and engaged in social problem-solving, goal setting, and peer interaction. Counsellors communicated biweekly with teachers to tailor support based on classroom performance. Recreational periods included sports and cooperative games to practice social skills in real-life contexts. A “behaviour call” system provided immediate feedback on prosocial and antisocial behaviours to reinforce targeted behavioural improvements.
Evans et al. (2014)	Combined	Challenging horizons programme – coaching	Yes	Yes	SMHP, other practitioner	39.87	Ten sessions for parents to create home behaviour contracts and use the Homework Management Plan. Adolescents completed a three-phase intervention: Phase 1, taught problem-solving vocabulary and guided creation of self-defined “self” goals. Phase 2, they practiced aligning behaviour with these goals in group social tasks and learned to interpret peer feedback. Phase 3, extended goal-driven behaviour to real-life interactions with adults, peers, and strangers, with structured feedback discussions.
Evans et al. (2016)	Combined	Challenging horizons after- school program	No	No	Student	121.05	Five core activities: primary counsellor meetings, an interpersonal skills group (ISG), recreation, an education/study skills group, and individual homework time. A behavioural level system guided feedback, using daily behaviour and teacher reports. Interventions included direct instruction in organisation, study skills, note-taking, and writing, with in-program practice and real-life application. ISG focused on identifying personal social goals and evaluating goal-consistent behaviour with counsellor support to enhance peer and adult interactions.
Evans et al. (2016)	Study Skills	Challenging horizons program– mentoring	No	No	Teacher	5.46	Mentors built supportive relationships while delivering interventions targeting academic (organisation, study skills, problem-solving) and social impairment (ISG, social problem-solving). Students set “ideal self” goals, practiced aligned behaviours, reviewed progress, and planned future interactions. Parents attended parallel sessions, received ADHD psychoeducation, and created behaviourally enforced homework plans.
Evans et al. (2023)	Combined	Multicomponent challenging horizons program	Yes	Yes	Graduate students as coaches, supervised by two psychologists	15	Interventions for academic impairment included organisation, problem solving, and study skills. Social impairment was addressed with individual and group sessions and social problem solving. In the first of 10 group sessions, students explored “ideal self” and “real self,” developed self-goals, practiced goal-aligned behaviour in activities, and reviewed progress with staff. Individual sessions focused on reviewing social events and planning future interactions based on ideal self-goals. Parents attended 10 parallel sessions, received ADHD psychoeducation, and created behaviourally enforced homework management plans with flexible structure.
Fabiano et al. (2010)	Daily Report Card	Daily report card	Yes	Yes	Teacher	NR	Consultants met with each teacher to design an individualised Daily Report Card (DRC) based on goals and related data. Teachers implemented the DRC, with behaviour targets refined at a second visit using collected data. A third visit fine-tuned the DRC and aligned it with home-based rewards. The DRC, completed daily by teachers, tracking ADHD-related target behaviours and goals, with in-day feedback provided to students. Parents attended three training sessions, learned to link home rewards to DRC outcomes, and developed tiered reward menus based on daily performance.
Haack et al. (2021)	Combined	Collaborative life skills-FUERTE	Yes	Yes	SMHPs	6	Students received continuous prompting and reinforcement for school, family, and social goals. Group sessions teaching organisation and social skills (e.g., routines, good sportsmanship, handling teasing). Skills were taught through didactic instruction, modelling, interactive games, role-play, and feedback. Reinforcers included praise, “stars,” and small prizes. Attention checks supported self-management, and generalisation was encouraged by exchanging home/school stars for group rewards. Behaviour goals were tracked via daily report cards rated by teachers and reviewed with students, with tailored academic and social targets reinforced across settings to support skill transfer.
Jurbergs et al. (2010)	Daily Report Card	DRC with parent consequences	Yes	Yes	Teacher	NR	Students participated in a structured daily behaviour report card (DBRC) intervention. Each morning, they placed the card on their desk for teachers to complete during the day by rating target behaviours (“yes,” “so-so,” or “no”). Teachers also marked smiley faces off for off-task behaviour and reviewed ratings privately with the student using a set script. After school, students took the DBRC home, reviewed it with a parent, calculated points, and, if sufficient points were earned, selected a reward from a pre-generated list. Students helped choose rewards to enhance motivation and were taught the system via instruction and rehearsal.
Jurbergs et al. (2010)	Daily Report Card	DRC without parent consequences	No	Yes	Teacher	NR	Each morning, students placed a behaviour report card in a designated folder on their desks. Teachers rated target behaviours using “yes,” “so-so,” or “no,” and had students cross off a smiley face for each off-task behaviour. Before lunch, teachers reviewed ratings privately with students using a structured script. The form was returned to the folder by the student. Prior to implementation, students were taught the procedure during a 15-min session with instruction, modelling, and rehearsal. This daily process emphasized student awareness and accountability for their classroom behaviour.
Jurbergs et al. (2010)	Daily Report Card	DRC with teacher consequences	Yes	Yes	Teacher	NR	Authors could not find any information on this group in the paper. Rest of the information about the intervention was taken from Moore et al. (2018).
Khilnani et al. (2003)	Relaxation	Massage therapy	No	Yes	Other	2.66	In a special education school, each student in the massage therapy group received two 20-min massages per week for a total of nine treatment sessions.
Lan et al. (2018)	Cognitive training	Executive Function Training	No	Yes	Psychologist and a psychology trainee	12	Children received training in two integrated parts: computerised cognitive tasks and group-based games incorporating executive function (EF) components and metacognitive skills. Group sessions followed structured rules such as taking turns, active listening, respectful self-expression, and emotional sensitivity. Each session targeted core EF areas: working memory, inhibition, flexibility, and planning. At the end of each session, therapists guided children in developing metacognitive skills by reflecting on problem-solving strategies. Children were rewarded with small gifts for positive participation and progress.
Lan et al. (2018)	Social skills training	Social skills training	No	Yes	Therapists	12	Training focused on enhancing social competence through structured sessions including homework review, didactic lessons, behavioural rehearsal, and coached play. Therapists used a token system to reinforce positive behaviours. Intervention modules included social behaviour, social cognition, and emotion regulation. Children received feedback during sessions, and parents were instructed to prompt and support the use of social skills at home to encourage generalisation across settings.
Langberg et al. (2012)	Study skills	Homework, organisation, and planning skills (HOPS)	Yes	Yes	SMHP	5.18	Targeting organisation, homework management, and time-management through 20-min sessions, initially twice weekly. Skills taught were for organising materials and recording homework, followed by planning and time-management. Students earned points for meeting specific checklist criteria (e.g., no loose papers, test recorded in planner) and exchanged them for gift cards. After 10 sessions, focus on problem-solving and self-monitoring. Two parent meetings supported skill generalisation at home, teaching parents how to track checklist completion and implement the point system after the intervention ended.
Looyeh et al. (2012)	Combined	Narrative therapy	No	No	SMHP	12	Six group activities for story-making, storytelling and guiding the children through the narrative process. The activities were a collection of play therapy activities, which were repeated in more than one session, with various stories directed at different symptoms, behaviours, and outcomes.
Mikami et al. (2021)	Combined	Making Socially Accepting Inclusive Classrooms (MOSAIC)	No	Yes	Teachers, trained by consultants	8.7 h on average teachers’ training, session items applied throughout school year	Teachers received a manual and orientation on strategies targeting deficient behaviours and negative peer dynamics that contribute to poor outcomes for children at risk for ADHD. They participated in structured consultation sessions, received feedback based on bi-monthly classroom observations, and collaboratively, with consultants, planned future implementation to support effective use of strategies that promote behavioural improvements and inclusive peer interactions.
Nejati et al. (2023)	Cognitive training	AMIN: Active memory intervention	Yes	Possibly Yes	NR	NR	Includes 21 progressive, paper-and-pencil tasks targeting all components of a working memory model. Tasks were designed around specific component functions, increasing in difficulty across 20 stages. Each task was presented in auditory/visual modalities, and children’s accuracy and speed were recorded on a worksheet to monitor progress and performance throughout training.
Omizo and Michael (1982)	Neurofeedback	Biofeedback- induced relaxation training	No	Yes	SMHP	1.66	Authors could not access this article; rest of the information was taken from Moore et al. (2018).
Pfiffner et al. (2016)	Combined	Collaborative life skills	Yes	No	SMHP	21	The child component involved nine group sessions during school hours, focusing on social functioning and independence. Skills taught included sportsmanship, problem solving, self-control, homework completion, and routine management. Sessions used instruction, rehearsal, and real-life practice, with age-appropriate activities encouraging leadership for older children. A reward-based system reinforced participation and skill use. Children earned tokens and rewards for meeting goals at school and home, promoting generalisation. Parent and teacher meetings supported refining target behaviours, coordinating homework plans, and reinforcing skills across settings.
Rivera and Omizo (1980)	Neurofeedback	Biofeedback- induced relaxation training	No	Yes	NR	0.4	Authors could not access this article; rest of the information was taken from Moore et al. (2018).
Seeley et al. (2009)	Combined	First step to success	Yes	Yes	Teacher, other practitioner	NR	Coordinated support across school and home to help children develop key prosocial and school-readiness skills. In the classroom, children were guided to improve cooperation, communication, and friendship-making. During six weekly home visits, parents were trained to teach their child sharing, problem-solving, limit setting. Using instruction, role playing, prompting, and feedback, parents learn how to embed these skills in everyday interactions and collaborate with teachers to reinforce their use at school.
Sibley et al. (2018)	Combined	High intensity summer intervention	Yes	Yes	School district personnel, SMHPs	360	Sessions included small and large group modules focused on organisation, time management, homework, study skills, social pragmatics, and vocational tasks. Verbal feedback on positive behaviours. Contingency management motivated adolescents through daily goals, privileges, and social events. Parent training used a community-based model with small and large group discussions, emphasising academic monitoring, routines, and parent-teen contracts.
Sibley et al. (2018)	Combined	Low intensity summer intervention	Yes	Yes	School district personnel, SMHPs	12	Organisational skills group, combining didactic instruction, hands-on activities (e.g., organising a backpack with peers), and group discussions. Youth practiced skills at home, such as organizing and scheduling, with plans coordinated during brief parent-youth meetings. Parents attended simultaneous group sessions, using the community-based model to reinforce skills and monitor progress throughout the transitional school year.
Smith et al. (2017)	Combined	Integrated Brain, Body and Social (IBBS) intervention	No	No	Classroom teachers, mental health professionals	120	Combined computerized cognitive training, physical exercise, and a classroom-based behaviour management strategy to target core cognitive deficits in ADHD. Computer tasks develop attention, working memory, and inhibition through progressively difficult exercises similar to Go/No-Go and card-sorting tasks. Physical activities (e.g., agility ladder, juggling) enhance cognitive functions via coordinated movement. The social component uses a response-cost version of the Good Behaviour Game during sessions to reinforce appropriate behaviour. Training difficulty adjusts to individual progress gradually from single and simple to multiple and more complicated. Aims to strengthen supporting brain networks and reduce ADHD symptoms across cognitive, physical, and social domains.
Steiner et al. (2011)	Neurofeedback	Neurofeedback	No	Yes	Student	24	Using EEG sensors in a bike helmet to detect theta (4–8 Hz) and beta (12–16 Hz) brainwave activity, during which children play a game where focused attention causes an airplane to rise. Sessions use individualised baselines and provide real-time auditory and visual feedback, helping children improve attention as they progress to higher difficulty levels.
Steiner et al. (2011)	Cognitive Training	Standard computer format	No	Yes	Student	24	A range of visual and auditory exercises designed to reduce impulsivity and increase attentiveness to the presented task. The participants in this study used attention training and working memory modules.
Steiner et al. (2014)	Neurofeedback	Neurofeedback	No	Yes	Researcher	30	Intervention trained children to reduce theta-to-beta EEG ratios using a helmet with embedded dry sensors. As focus increased, participants advanced in exercises, for example, guiding a dolphin to collect coins. Distraction reversed progress. The system rewarded sustained attention.
Steiner et al. (2014)	Cognitive Training	Cognitive training	No	Yes	Researcher	30	Intervention used a standardised protocol of 14 age-appropriate auditory and visual exercises targeting attention, working memory, visual tracking, reaction time, and inhibition control. Tasks automatically progressed in difficulty and rewarded correct responses (e.g., unlocking a safe). Exercises rotated regularly, allowing scalable delivery.

Some core practices repeatedly emerged across interventions, highlighting a shared approach in addressing ADHD difficulties in children. As discussed in the literature (Richardson et al., 2015), intervention components varied greatly, combining both classroom management strategies and skills training. The majority of the interventions in our review adopted a skills-based model that aimed to equip children with specific cognitive, organisational, behavioural, academic, and social competencies to manage ADHD symptoms and related difficulties. Providing children with guided feedback, prompting, encouraging active participation and role-playing, setting and monitoring target behaviours, and reflection were key components across interventions. Classroom-based strategies (e.g., daily report card, preferential seating, and use of praise) and parent training were also commonly incorporated. Interventions were typically delivered in a structured format, through didactic teaching, reinforcement (e.g., token economies or point-based reward systems), and practice. Some cognitive interventions also emphasised performance-based progression, emphasising developmental sensitivity and tailored approach to learning and change. Centered around social skills, organisation and planning, and neurocognition, strategies were predominantly child-contingent for enduring and transferable skill-building. More detailed information on intervention components is included in Table 3.

### Study outcomes

All included studies reported core ADHD outcomes; ADHD (*k* = 22), inattention (*k* = 16), and hyperactivity/impulsivity (*k* = 16). Some studies also reported ADHD-accompanying impairments such as difficulties in academic skills (*k* = 14), social skills (*k* = 13), and externalising problems (*k* = 9). Measurements for each outcome are summarised here in brief and detailed information is provided in Table 4. Any measurement taken from Moore et al. (2018) was retained for consistency.

**Table 4:** Scales per study per outcome.

Study name	ADHD-C	R	IN	R	HI	R	AC	R	SS	R	EX	R
Bloomquist et al., 1991			CTRS Inattention	T	CTRS Impulsivity	T	Percent on task	O	Walker- McConnell Scale of Social Competence and School Adjustment	T	CTRS Conduct Problems	T
Capodieci et al., 2017			IPDDAI/IPDDAG	T, P	IPDDAI/IPDDAG	T, P	IPDDAI/IPDDAG – Working Memory	T				
Chacona, 2008			TOVA: Omission Auditory	T	TOVA: Commission Auditory	T						
Cloward, 2003	ADHD Index/DSM 5	T	Conners/DSM Inattentive Scale									
Egeland et al., 2013	ARS-IV Total	P, T	CPT-2 Focused attention/ARS-IV Attention	C, P, T	CPT-II Hyperactivity- Impulsivity/ ARS-IV Hyperactivity	C, P, T	Maths	C				
Evans et al., 2011			DBD	P, T	DBD	P, T	IRS	T				
Evans et al., 2014			DBD	P	DBD	P	IRS/CPS	P, T	IRS - Relationship with peers/CPS -Interpersonal performance	P, T		
Evans et al., 2016			DBD -Inattention	P, T	DBD –Hyper/impulsive	P, T	COSS - Task planning/% assignments turned in	P, T	SSIS/Impairment Rating Scale- relation with peers	P, T	DBD–ODD	P, T
Evans et al., 2023			ADHDRS−5	P	ADHDRS−5	P			SSIS	P, C	DBD–CD	P
Fabiano et al., 2010	DBD— ADHD	T P, T					WJ- Reading/APRS—Academic Success	C, T	Student- Teacher Relationship Scale	T	DBD – ODD/CD	T
Haack et al., 2021	CSI-4	P, T							IRS	P, T	CSI-4	P, T
Jurbergs et al. (2010)	Conners’ Rating Scale- Revised: Short Form	P, T					% work complete	O				
Khilnani et al. (2003)			CTRS	T	CTRS	T					CTRS	T
Lan et al. (2018)			ADHD- RS-IV/ CPT-II: Conners’ Continuous performance tasks- II- omissions	C, O	ADHD- RS-IV/CPT-II: Conners’ Continuous performance tasks- II- commissions	C, O	Working memory capacity	O	SAICA- Interaction with peers	P		
Langberg et al., 2012			Vanderbilt	T	Vanderbilt	T	COSS- Organisation-Task planning/COSS- Math Task Planning	P, T	COSS- Impairment- Family Conflict	P	COSS- Impairment-Life Interference	P
Looyeh et al., 2012	CSI-4	T	CSI-4	T	CSI-4	T						
Mikami et al., 2021	ADHD-5	T					ASF Enablers	T	Social preference sociometric / CLM Teacher support	C, T		
Nejati et al. (2023)	Conners-3	P, T					Executive Functions test- 1 back	O				
Omizo and Michael (1982)			MFFT attention		MFFT impulsivity	C						
Pfiffner et al., 2016	CSI: ADHD	P, T					COSS total	P, T	SISS social skills	P, T	CSI: ODD	P, T
Rivera and Omizo, 1980			MFFT attention	C	MFFT hyperactivity	C						
Seeley et al., 2009			SSRS- INATT	T	SSRS- HYP	T	SSRS-AC	T	SSBD-ABI/SSRS-SS	T, P	SSRS-PB/ TRF- ODD	P, T
Sibley et al., 2018			DBD	P, T	DBD	P, T	AAPC/Non- standardised Academic scales (Bookbag organisation and percentages on a checklist)	P, T, O	Conflict Behaviour Questionnaire-20 (Parent-teen conflict)	P, O		
Smith et al., 2017	SNAP/CGI Improvement	O, P, T					CVLT- Total learning	O				
Steiner et al., 2011	Conners- Revised ADHD Index	C, P, T	Conners- Revised Inattention	C, P, T	Conners- Revised Hyperactivity	P, C, T	BRIEF	P, T				
Steiner et al., 2014	SKAMP Total/Conners–3	T	Conners–3/SKAMP Attention	P, T	Conners 3P Hyperactivity/SKAMP Deportment	P, T	BRIEF	P	Conners −3- Peer Relations	P, T	Conners 3 T Aggression	P, T

Category abbreviations included in the table are IN–Inattention, HI–Hyperactivity, AC – Academic, SO – Social, EXP – Externalising Problems; R – Reporter, which were shortened as: P for Parent, T for Teacher, C for Child, and O for Observer. Full name abbreviations of the scales are included in the full text, but also here, as follows: Academic Competence Evaluation Scale (ASF Enablers), Academic Problems Rating Scale (APRS), Adolescent Academic Problems Checklist (AAPC), Attention-Deficit Hyperactivity Disorder Rating Scale-4 (ARS-4), Behaviour Rating Inventory of Executive Functions (BRIEF), California Verbal Learning Test (CVLT), Child Symptom Inventory (CSI), Children’s Organisational Skills Scales (COSS), Classroom Life Measure (CLM), Classroom Performance Survey (CPS), Clinical Global Impression Scale (CGI), Conners’ Teachers Rating Scale (CTRS), Continuous Performance Test (CPT), Disruptive Behaviour Disorders (DBD), Early Identification of ADHD (IPDDAG-IPDDAI), Homework Problems Checklist (HPC), Impairment Rating Scale (IRS), Matching Familiar Figures Test (MFFT), Problem Behaviour (PB), Social Adjustment Inventory for Children and Adolescents (SAICA), Social Skills Improvement System (SSIS) and Swanson, Kotkin, Agler, M-Flynn, and Pelham Scale (SKAMP), Swanson, Nolan and Pelham Scale (SNAP), Systematic Screening for Behaviour Disorder (SSBD) – Adaptive Behaviour Index (ABI), Social Skills Rating System (SSRS), Teacher Report Form (TRF), Test of Variables of Attention (TOVA).

#### ADHD

A wide range of scales was used to assess ADHD: Attention-Deficit Hyperactivity Disorder Rating Scale-4 (*k* = 2), Attention-Deficit Hyperactivity Disorder Rating Scale-5 (*k* = 1), Child Symptom Inventory-4 (*k* = 3), Conners Rating Scale (*k* = 6), Disruptive Behaviour Disorders (*k* = 5), Matching Familiar Figures Test (*k* = 2), ADHD-5 (*k* = 1), Clinical Global Impression Scale (*k* = 1), Continuous Performance Test (*k* = 1), Early Identification of ADHD (*k* = 1); Swanson, Kotkin, Agler, M-Flynn, and Pelham Scale (*k* = 1); Swanson, Nolan and Pelham Scale (*k* = 1), Social Skills Rating System (*k* = 1), Test of Variables of Attention (*k* = 1), and Vanderbilt (*k* = 1).

A higher number of studies (*k* = 8) had ADHD-combined as the dominant subtype within their samples, followed by inattention (*k* = 3) and hyperactivity (*k* = 2). However, the majority of studies (*k* = 13) did not report this finding. For the use of ADHD stimulants across control and treatment groups, of 26 included studies, only 4 explicitly excluded children on medication, 6 did not report medication status, and 20 included children on medication at varying levels.

#### Academic skills

Academic skills were measured using a wide range of scales including standardised tests, grades, and non-standardised measures. Cognitive skills, e.g., executive function and working memory, were also categorised under the academic skills as they highly positively correlated. In terms of standardised measures, the following scales were used: Academic Competence Evaluation Scale-short form (*k* = 1), Academic Performance Rating Scale (*k* = 1), Adolescent Academic Problems Checklist (*k* = 1), Behaviour Rating Inventory of Executive Functions (*k* = 2), California Verbal Learning Test (*k* = 1), Conners’ Classroom Performance Survey (*k* = 1), Continuous Performance Test (*k* = 1), Early Identification of ADHD-Working memory subscale (*k* = 1), Homework Problems Checklist (*k* = 1), Impairment Rating Scale (*k* = 1), Social Skills Rating Scale-Academic subscale (*k* = 1), and Children’s Organisational Skills Scales (*k* = 1). Additionally, the following non-standardised measures and school grades were used: percentage of assignments turned in or work completed (*k* = 3), math and reading scores (*k* = 2), executive function (*k* = 1) and working capacity (*k* = 1).

#### Social skills

While some studies reported negative aspects of social skills (e.g., social skills impairments), some others focused on positive aspects (e.g., social skills improvement). Measures reporting children’s social skills impairments were as follows: Impairment Rating Scale (*k* = 3), Children’s Organisational Skills Scale-Impairment-Family conflict (*k* = 1), Classroom Performance Survey-Interpersonal performance subscore (*k* = 1), Classroom Life Measure-Teacher support (*k* = 1), Conflict Behaviour Questionnaire–20–Parent-teen conflict (*k* = 1), Conners–Peer Relations (*k* = 1) and Asocial subscore (*k* = 1). Scales that focused on social skills improvement were: Social Adjustment Inventory for Children and Adolescents-Interaction with peers subscore (*k* = 1), Social Skills Improvement System (*k* = *3*), Social Skills Rating System (*k* = *1*), Social preference sociometric measures (*k* = 1), Student-Teacher Relationship Scale (*k* = 1) and Walker-McConnell Scale of Social Competence and School Adjustment (*k* = 1).

#### Externalising problems

Standardised measures were used to assess varying aspects of externalising problems such as aggression, disruptive behaviours, conduct problems and oppositional defiant disorder. Specific measures to report children’s externalising problems were as follows: Conners Teacher Rating Scale (*k* = 3), Disruptive Behaviours Disorder Rating Scale (*k* = 3), Child Symptom Inventory-4 (*k* = 2), Children’s Organisational Skills Scale-Impairment-Life Interference (*k* = 1), Social Skills Rating Scale (*k* = 1) and Teachers’ Report Form (*k* = 1).

### Quality appraisal

Quality appraisal of pre-2018 studies were gathered from Moore et al. (2018) and post-2018 studies were evaluated by the first author. Of the included studies, 6 showed high risk of bias, 12 showed some concerns, and 8 showed low risk of bias. There were some concerns regarding randomisation procedure and allocation concealment as only few studies reported these. In regards to blinding, there were again some concerns as in most studies (*k* = 20) the assessors were aware of participants’ assigned groups. Furthermore, intervention fidelity appeared to be good across most of the included studies as 19 out of 24 studies had above 85% intervention fidelity, while two papers did not report this finding or students’ attendance. Additionally, only a small number of included studies (*k* = 9) shared follow-up results. Finally, only 12 out of 26 studies reported missing data and how it was handled. Detailed information regarding quality appraisal can be seen in Table 5.

**Table 5:** Risk of bias analysis.

Study details	Randomisation specified	Allocation concealment	Blinding	Response rate/Fidelity/ attendance	Follow up	Missing data	Selective reporting	Risk of bias result
Bloomquist et al. (1991)	N	N	Y	<70%	Y	Y	Y	Some concerns
Capodieci et al. (2017)	Y- Class level clustered	N	Y–Delivery N–Assessor	85%+	N	Y	N	Low risk
Chacona (2008)	Y	Y	N	85%+	N	NA	Y	Low risk
Cloward (2003)	N	N	N	85%+	N	NA	N	Some concerns
Egeland et al. (2013)	Y	Y	N	85%+	Y	Y	Y	Low risk
Evans et al. (2011)	N	N	N	NR	N	N	Y	High risk
Evans et al. (2014)	N	N	N	70–84%	N	Y	Y	High risk
Evans et al. (2016)	N	N	N	85%+	Y	Y	Y	Some concerns
Evans et al. (2023)	Y- Stratified based on medication and sex, school level randomisation	N	N	85%+	Y	N	N	Low risk
Fabiano et al. (2010)	N	N	Y	85%+	N	Y	Y	Some concerns
Haack et al. (2021)	Y- 2-level cluster	Y	N	85%+	N	N	Y	Low risk
Jurbergs et al. (2010)	N	N	N	85%+	N	NA	Y	Some concerns
Khilnani et al. (2003)	N	N	N	85%+	N	N	Y	Some concerns
Lan et al. (2018)	N	Y	Y	NR	N	NR	Y	Some concerns
Langberg et al. (2012)	N	N	N	85%+	Y	N	Y	Some concerns
Looyeh et al. (2012)	N	N	Y	85%+	Y	NA	Y	Low risk
Mikami et al. (2021)	N	N	N	80%+	N	Y	Y	High risk
Nejati et al. (2023)	N	N	NR	NR	Y	N	Y	High risk
Omizo and Michael (1982)	Y	N	N	85%+	N	NA	Y	Some concerns
Pfiffner et al. (2016)	N	N	N	85%+	N	Y	Y	High risk
Rivera and Omizo (1980)	Y	N	N	85%+	N	NA	Y	Some concerns
Seeley et al. (2009)	N	N	N	85%+	N	NA	Y	Some concerns
Sibley et al. (2018)	N	N	N	85%+	Y	Y	Y	Some concerns
Smith et al. (2017)	Y-Stratified based on medication	Y	Y	80%	N	Y	Y	Low risk
Steiner et al. (2011)	N	N	N	85%+	N	Y	Y	High risk
Steiner et al. (2014)	Y	N	Y	85%+	Y	Y	Y	Low risk

This scale is an adapted version of the Cochrane risk of bias tool included in Moore et al. (2018) study. The following revisions were made on this table: 1. “Intention to Treat” and “6 month follow up” columns were removed. 2. New studies after 2018 were added accordingly but no changes were made on Moore et al. (2018)‘s initial analysis. 3. A column for High risk–some concerns–low risk was added to the table. Following their reporting style, the studies were rated in the format of Yes, No, No Response or Not Applicable.

### Meta-analytic findings

In total, 22 unique studies were meta-analysed. Some studies were included more than once for certain outcomes due to having different reporters (e.g., parents, teachers, self-reports). Hence, total number of studies included in each meta-analysis were as follows: ADHD (*k* = 35), inattention (*k* = 25), hyperactivity/inattention (*k* = 25), academic skills (*k* = 22), social skills (*k* = 19), externalising problems (*k* = 12). For each meta-analysis, we checked the presence of publication bias. Visual inspection of funnel plot asymmetry raised concerns over potential publication bias for ADHD and inattention outcomes which was also confirmed by Egger’s test findings: ADHD (*t* = –3.12, *df* = 33, *p* < 0.01), inattention (*t* = –2.41, *df* = 23, *p* = 0.024). Detailed findings for publication bias can be seen in Supplementary Figures S2–S7.

#### ADHD

**Figure 2:**
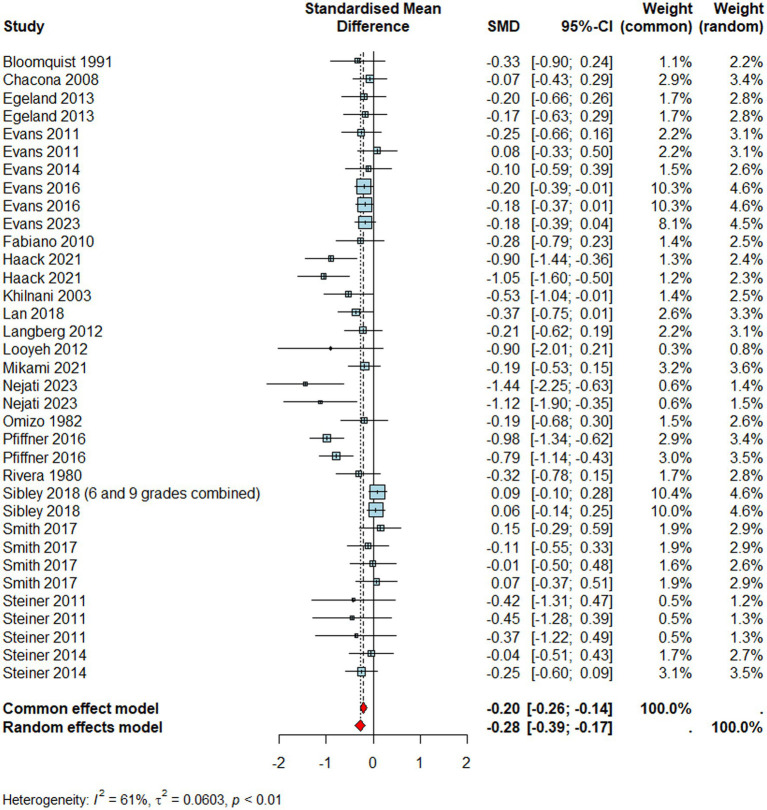
Forest plot for ADHD.

Pooling effect sizes across studies reporting ADHD outcomes (Figure 2, *n* = 4256) resulted in a small to medium and significant negative effect size (*d* = –0.28, 95% CI [–0.39, –0.17], *p* < 0.0001) suggesting that children with ADHD who received a school-based intervention showed significantly lower levels of ADHD than those in the control conditions. Meta-regression analysis revealed that while the type of rater did not explain a significant amount of variance in the pooled effect size (*Q*_M_ = 3.03*, p >* 0.05), interventions were more effective in primary than secondary school settings (*Q*_M_ = 5.34, 𝑝 = 0.021). However, the pooled effect size suffered from significant heterogeneity for ADHD outcomes (*I^2^ =* 61%*, τ^2^* = 0.06, *p <* 0.01) which may suggest these interventions may not share a common effect size.

#### Inattention

**Figure 3:**
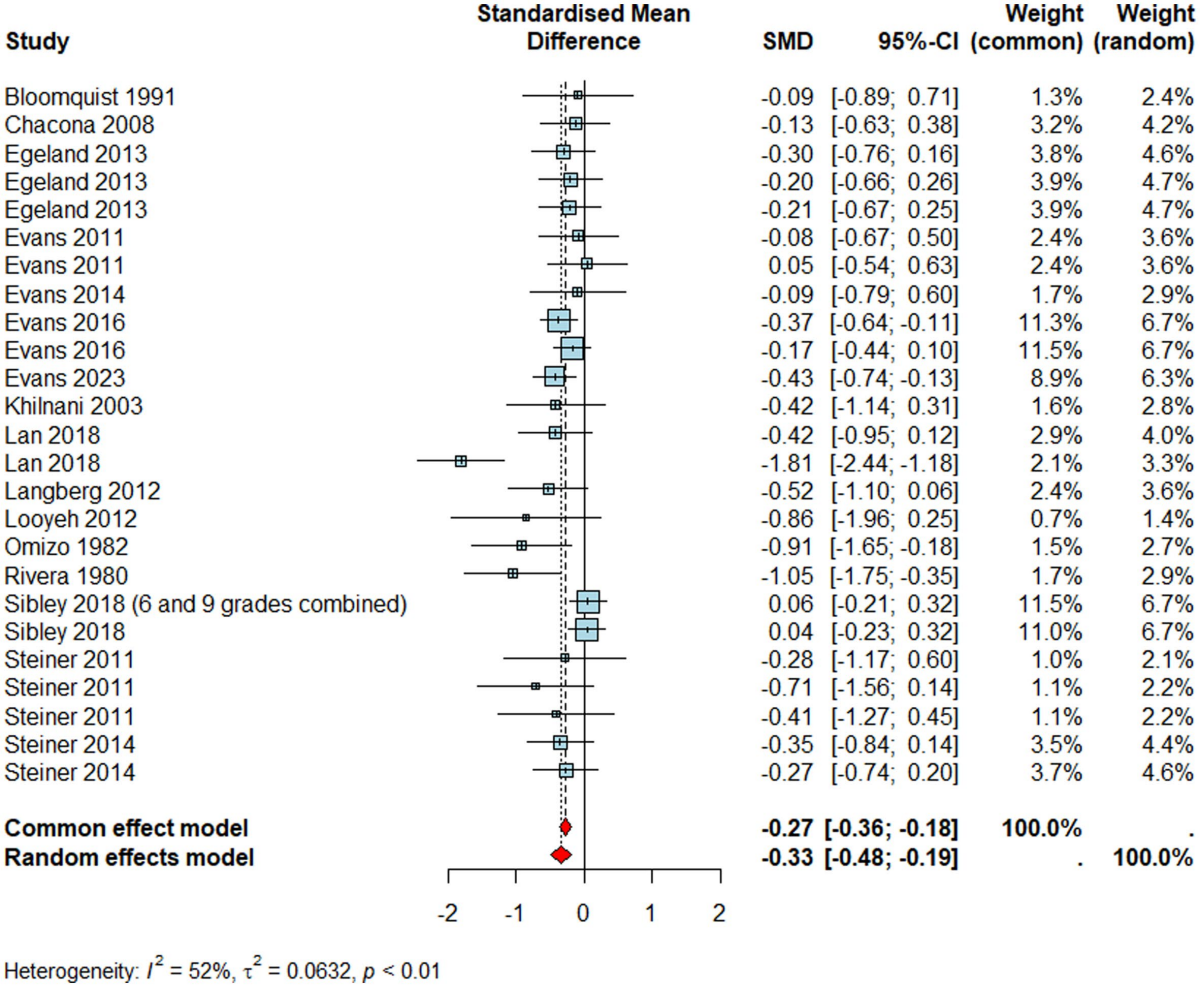
Forest plot for inattention.

Studies reporting inattention outcomes (Figure 3, *n* = 1933) resulted in a medium negative effect size (*d* = –0.33, 95% CI [–0.48, –0.19], *p* < 0.0001) suggesting improvements in inattention in children who were exposed to a school-based intervention than those who were not. However, meta-regression findings revealed that reporter type significantly moderated the effectiveness of interventions (*Q*_M_ = 27.16*, p <* 0.001). Specifically, compared to the child reports (i.e., reference category), teachers (*β* = 0.47, *p* = 0.014, 95% CI [0.10, 0.84]) and parents (*β* = 0.41, *p* = 0.030, 95% CI [0.04, 0.79]) perceived significantly less improvements in children's inattention symptoms post-intervention. On the contrary, observers reported significantly higher gains in inattention symptoms than child reports (*β* = −1.17, *p* = 0.002, 95% CI [−1.91, −0.43]), although this suffered from power issues as only one outcome was observer-reported. Similar to the combined ADHD outcome, interventions targeting primary school-aged children were significantly more effective to improve attention skills than those delivered in secondary school settings (*Q*_M_ = 4.55, 𝑝 = 0.03). There was also significant heterogeneity (*I^2^* = 52%*, τ2* = 0.06*, p* < 0.01) which may indicate that such trials may not share a common effect size on inattention.

#### Hyperactivity/Impulsivity

**Figure 4:**
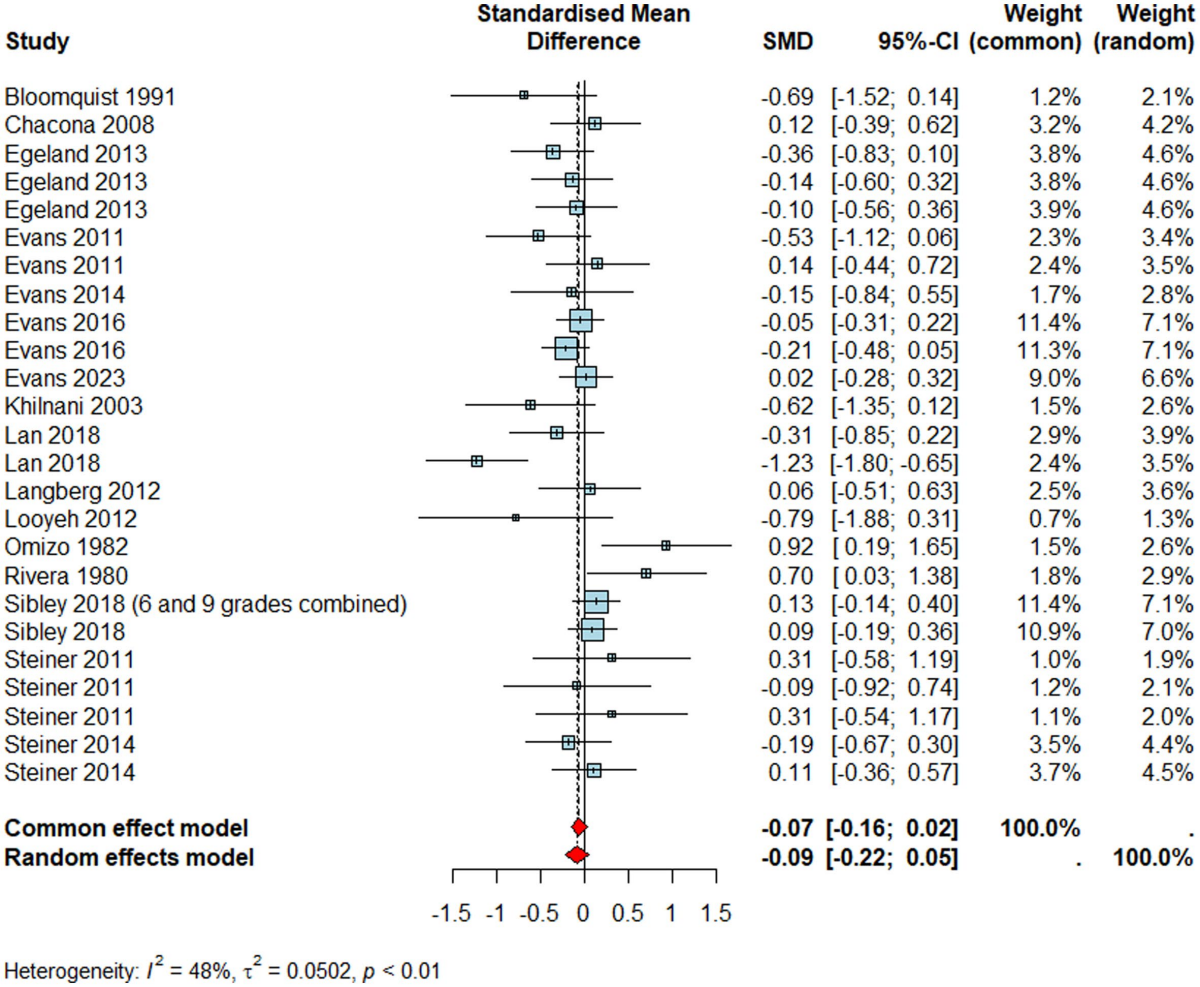
Forest plot for hyperactivity/impulsivity.

Studies reporting hyperactivity/impulsivity (Figure 4, *n* = 1933) showed no significant effects (*d* = –0.09, 95% CI [–0.22,0.05], *p* = 0.22) meaning that individuals who received school-based interventions did not improve their hyperactivity/impulsivity symptoms compared to those who did not. Similar to inattention, observers reported significantly greater treatment effects for hyperactivity symptoms (*Q*_M_ = 15.42, *p* < 0.01) compared to the reference group (i.e., child outcomes) (*β* = −1.39, *p* < 0.001, 95% CI [−2.09, −0.69]). However, it is important to bear in mind that there was only one observer-reported hyperactivity/impulsivity outcome included in the meta-regression analysis of hyperactivity, and parental or teacher reports did not statistically differ from the reference group.

#### Academic skills

**Figure 5:**
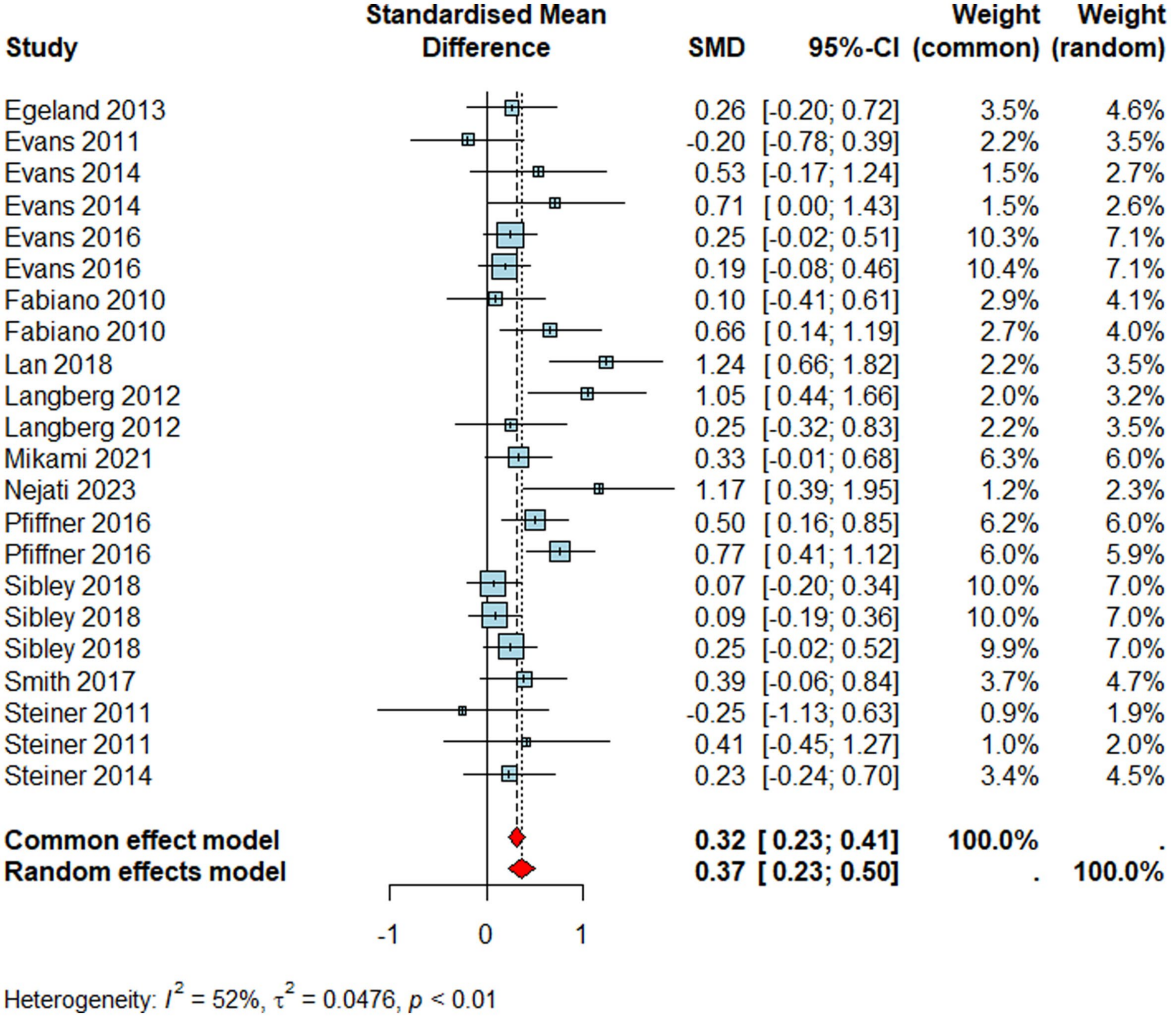
Forest plot for academic skills.

For studies reporting academic skills (Figure 5, *n* = 2138), we found a significant moderate positive effect size (*d* = 0.37, 95% CI [0.23 0.50], *p* < 0.0001), that is, individuals who received school-based interventions improved their academic skills beyond those who did not. Similar to the ADHD combined, reporter type did not moderate the pooled effect size (*Q*_M_ = 2.78, *p > 0*.05) and interventions appeared to be more effective in primary than secondary school settings (*Q*_M_ = 5.48, *p* = 0.01). Like other outcomes, the pooled effect size suffered from significant heterogeneity (*I^2^* = 52%*, τ^2^* = 0.05*, p* < 0.01).

#### Social skills

**Figure 6:**
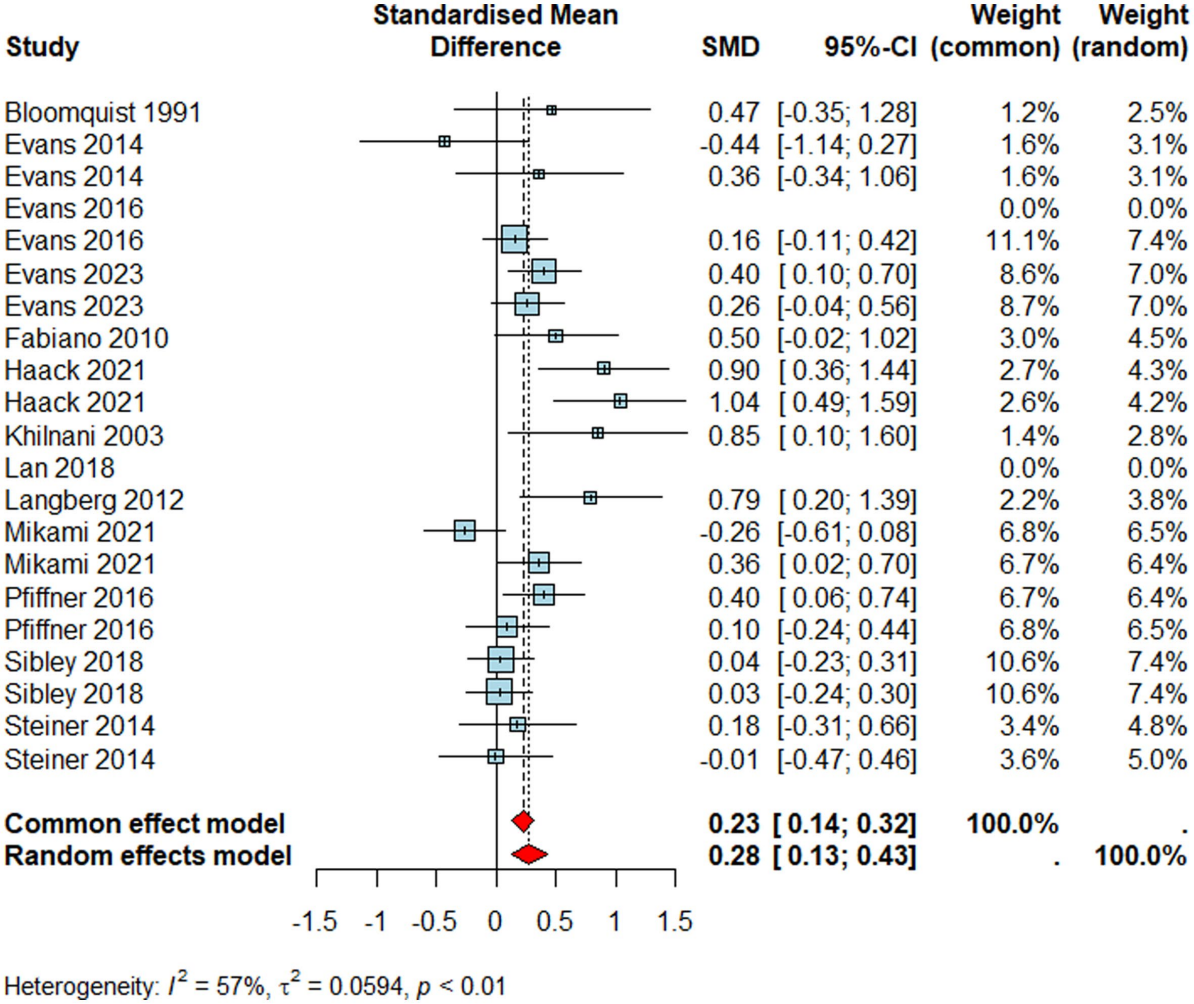
Forest plot for social skills.

Studies reporting social skills (Figure 6, *n* = 1991) revealed a significant small to medium positive effect size (*d* = 0.28, 95% CI [0.13 0.43], *p* < 0.001) meaning that individuals who received a school-based intervention improved their social skills more than those who did not. Neither the type of reporter (*Q*_M_ = 3.31*, p >* 0.05) nor the school-level (Q_M_ = 0.29, *p >* 0.05) moderated the effectiveness of school-based interventions on social skills. As others, trials reporting social skills outcomes also suffered from significantly high heterogeneity (*I^2^* = 57%*, τ^2^* = 0.06*, p* < 0.01).

#### Externalising problems

**Figure 7:**
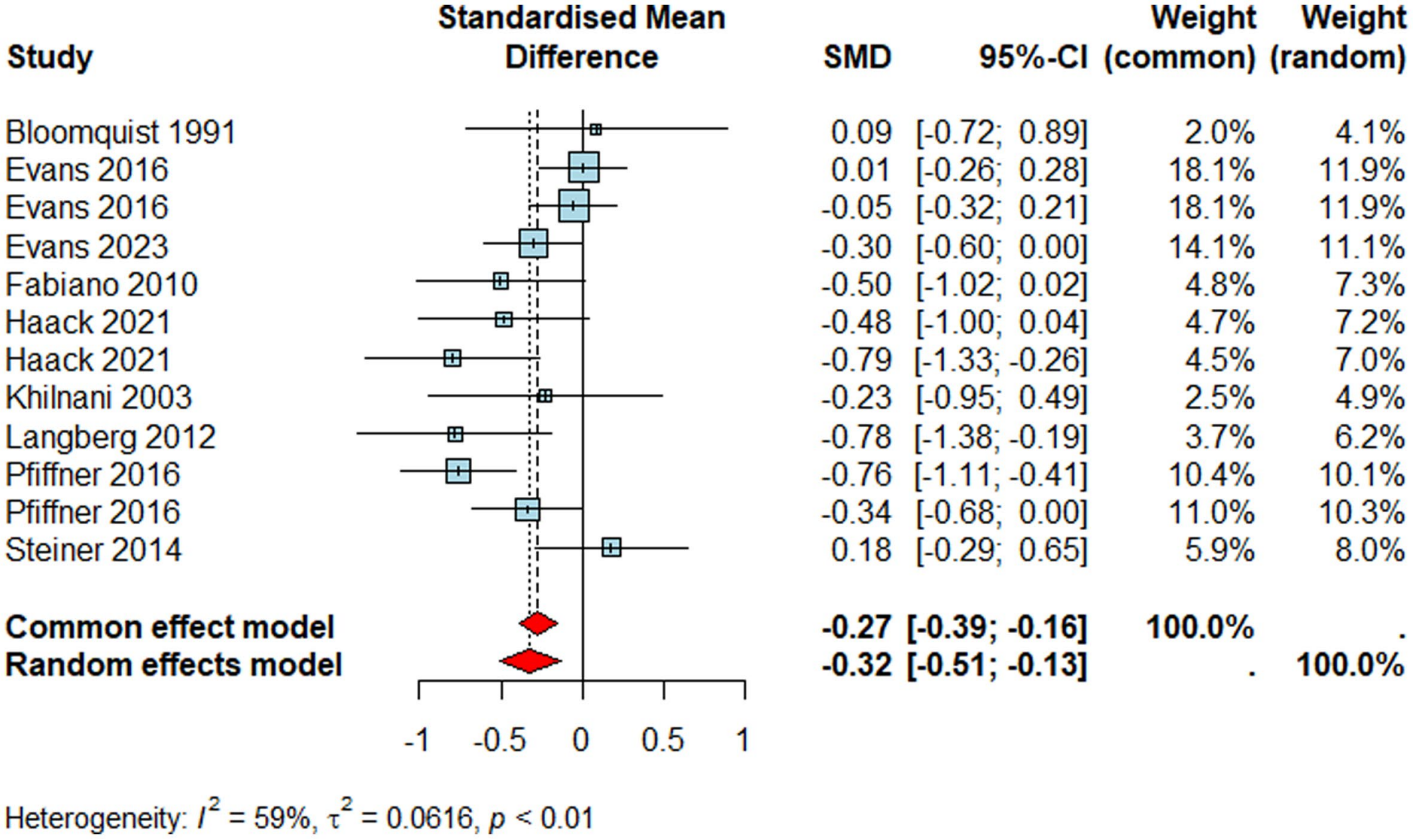
Forest plot for externalising problems.

Finally, studies reporting externalising problems (Figure 7, *n* = 1217) resulted in a negative, medium and significant effect size (*d* = –0.32, 95% CI [–0.51, –0.13], *p* = 0.001). That is, individuals who received a school-based intervention had lower levels of externalising problems than their peers who did not. Similar to social skills outcomes, the type of reporter did not moderate the pooled effect size (*Q*_M_ = 0.79*, p >* 0.05), same as the school type (*Q*_M_ = 1.19, *p >* 0.05). Finally, the pooled effect size for externalising problems also suffered from statistically high heterogeneity (*I^2^* = 59%*, τ^2^* = 0.06*, p* < 0.01).

## Discussion

The current paper systematically reviewed and conducted a meta-analysis of the effectiveness of school-based interventions (1980-2024) on core ADHD symptoms and accompanying impairments, including academic, social and externalising problems. Overall, 26 studies met the eligibility criteria, 22 of which were included in the meta-analyses. We found that school-based interventions were effective in reducing symptoms related to ADHD (combined), inattention, and externalising problems and improving academic and social skills in school-aged children with ADHD. Such interventions appeared to be more effective if delivered in primary school settings compared to secondary school settings, except for social skills and externalising problems, where school type did not make a difference on the observed effects. Additionally, school-based interventions appeared to be ineffective in reducing hyperactivity/impulsivity. To note, our findings suffer from high heterogeneity and some level of potential reporter-bias, which are two common issues in the field.

Interventions in this review involved a combination of school adjustments and skill-training for children, latter of which was emphasised more frequently across studies. That is, interventions prioritised and targeted equipping children with social, organisational, academic and behavioural strategies, encouraging child’s agency in managing ADHD difficulties through reinforcing feedback and reflection, setting and evaluating goals, providing clear instructions and prompting. Furthermore, our meta-analyses statistically indicated that school-based interventions are effective in improving core ADHD difficulties of school-aged children with ADHD, mainly the symptoms of ADHD (combined) and inattention. These findings align with previous reviews and meta-analyses, which also reported significant positive effect sizes for school-based interventions targeting core ADHD difficulties (Moore et al., 2018; DuPaul et al., 2012; DuPaul and Eckert, 1997). An interesting observation from our review, however, was that the level of change in hyperactivity for children in the intervention groups was not statistically lower than their peers in the control groups. A previous meta-analysis presented similar results, arguing that school-based psychosocial interventions might be more effective in addressing inattention problems than hyperactivity (Richardson et al., 2015).

There may be several reasons for why school-based interventions appear to be effective in improving inattention symptoms but not hyperactivity. It is recognised that inattention and hyperactivity are pathophysiologically distinct constructs (Willcutt et al., 2012). On this ground, we could argue that school-based interventions may be explicitly, or more so, focused on improving attention-related skills (e.g., organisational, homework) than hyperactivity difficulties. Findings from a recent meta-analysis also supports this argument by showing varied strength levels for the interventions’ effectiveness on hyperactivity across studies, such as social skill or cognitive/neurofeedback interventions that had limited power on improving impulsivity (Sadr-Salek et al., 2023). Perhaps, inclusion of more physical and relaxation-based components in the intervention could make management of hyperactive/impulsive symptoms easier. Another explanation for this might be the distribution of ADHD types in study samples, where hyperactivity always had the smallest percentage, except the two studies with 100% hyperactive ADHD groups. Naturally, the preponderance of inattention would mean there were more opportunities to notice the changes in these symptoms than hyperactivity. It is also plausible that children with predominantly hyperactive symptoms may be more likely to receive medication earlier compared to their peers with inattentive presentation, due to the visibility and disruptiveness of impulsive behaviours in the classroom, some of which might have already been addressed by medication. Therefore, introducing a school-based intervention might have only produced small gains for this group, resulting in a possible ceiling effect. Our analyses would not have captured the within-group differences in these children if there were any.

Combined effects of school-based interventions also appear to improve the studied ADHD-accompanying difficulties. The significant gains in ADHD-related impairments in our paper reinforces the earlier findings, where psychosocial classroom interventions were effective in enhancing academic performance and social skills and reducing conduct problems, including off-task behaviour (Huang et al., 2021; De Siqueira et al., 2019; Harrison et al., 2019; Daley et al., 2014; DuPaul et al., 2012; Fabiano et al., 2009; Miranda et al., 2006). Comparatively, Moore et al. (2018) showed mixed results for ADHD-related impairment outcomes based on the intervention types and reporters. For instance, there was a positive impact of multimodal interventions on academic skills across all raters, on conduct problems for parents’ ratings (included under externalising problems in our paper), but no statistically meaningful improvement in social skills of children in the treatment groups compared to control groups. Our meta-analyses revealed similar results for academic skills, as we found significant benefits of interventions regardless of the type of reporter. Our meta-analytic findings of externalising skills were significant too, yet independent of the reporter type. Finally, our meta-analysis of social skills also yielded significant results, contrary to Moore et al. (2018)‘s findings on this outcome, although the effect size was slightly lower than the other two outcomes in this domain. Taken together, our results indicate that the pooled school-based ADHD interventions from 1980 to 2024 are promising in addressing academic and social skills as well as externalising problems of children with ADHD.

We also conducted meta-regression analyses for school types. Our findings highlighted the importance of early interventions for ADHD combined, inattention symptoms, and academic skills, as school-based interventions for primary school-aged children were better at targeting these domains than the ADHD interventions in secondary schools. Additionally, meta-regression analyses based on each rater (i.e., teacher, parent, child, observer) were performed to evaluate the potential influence of the reporter type for all measured outcomes. While there was no significant moderator effect of the outcome reporter on ADHD combined, which is similar to Moore et al. (2018), there was noticeable influence of raters on the observed effects for inattention and hyperactivity. However, it is important to note that for inattention, parents and teachers did not report as much impact of interventions as children observed. For hyperactivity, none of the reporters perceived differences between treatment and control groups after the interventions, only that one observer moderated the overall effects by reporting lower gains from the intervention compared to the child reports. Past research, including the precedent meta-analysis, underlined the dependence of interventions’ effectiveness on the type of intervention and reporter (Moore et al., 2018; Watabe et al., 2013). While the problem of reporter-bias is prevalent in the field, inconsistency in reporters’ understanding of the problem and focus on different target behaviours might explain the disagreement (Hartman et al., 2007). This would mean that the observed tasks and behaviours to report on ADHD symptoms differ among raters, as shown in our results, where teachers and parents perceived less improvement in inattention following the interventions compared to the child. Combined with this finding, the majority of the measures in the included studies relied on subjective ratings, it might be that the interventions are teaching children how to compensate for their symptoms to meet the demands and expectations set by the school, parents, and peers, especially when they are unblinded to the intervention conditions. Interventions that enforce compliance, classroom behaviour management, or external reinforcement systems may inadvertently teach children to mask overt signs of inattentiveness without addressing underlying neurocognitive difficulties. As the efficacy of school-based interventions on academic, social, and conduct problems of ADHD-related impairment appeared to be stable and consistent across different raters, it is likely that interventions are equipping children with strategies to apply in the classroom that effectively target these skills, which are equally visible to others, such as parents and teachers. As a final note, while some studies included observational and experimental tasks, more so in academic and social skills than the core ADHD symptoms, the majority of data in our analyses were drawn from subjective assessments. As such, the differences in raters’ observations, especially in inattention ratings, should reflect the difference in reporters’ subjective measures. It is important for future studies to incorporate multi-informant and objective measures, such as neurocognitive testing alongside standardised, self-report data to support observed effects and reduce bias.

We should also consider the high levels of statistical heterogeneity for all ADHD and ADHD-related impairment outcomes. This remains to be a common issue in the field, as seen in previous reviews focused on ADHD (Moore et al., 2018; DuPaul et al., 2012; DuPaul and Eckert, 1997) and in other neurodevelopmental subgroups of the population (e.g., autism; Deniz et al., 2022, 2024; Francis et al., 2022). Taken together, while present school-based interventions are effective in improving ADHD accompanying difficulties, the variance between study or intervention characteristics in meta-analyses, such as the outcome reporter, outcome measurement, intervention provider, and duration of the intervention (e.g., the shortest intervention lasted 25 minutes), complicates the interpretation of the pooled effects. This complexity may also partly reflect the heterogeneity inherent to ADHD itself, which presents differently across individuals. As such, it is possible that tailored or individualised interventions are better suited to meet the diverse needs of children with ADHD, and that variability across interventions highlight the need to personalise support. This flexibility in practice, however, also contributes to inconsistencies in intervention designs that may limit the interpretability and generalisability of meta-analytic findings. Balancing the individualisation in practice with standardisation in research might be key to advancing both the effectiveness and applicability of school-based ADHD interventions.

### Strengths and limitations

This review and meta-analysis hold several strengths. First, following the PRISMA guideline in reviewing and reporting the existing evidence ensures no selective-reporting bias, which was further strengthened by taking the first-reported outcome by each rater per domain per study. Furthermore, inclusion of multiple reporters per outcome, wherever available, and performing meta-regression analyses also unpacked potential reporter-related bias, especially given that reporters were not blinded to child’s assigned study conditions in most cases, which triggers observer-expectancy effect. Third, while the inclusion of the grey literature is a clear strength, as it reduces the possibility of publication bias, it may as well introduce other types of bias due to inclusion of data that have not been rigorously peer reviewed. Fourth, inclusion of new interventions that were not studied by the previous meta-analysis (e.g., IBBS–Integrated Brain, Body and Social Intervention) as well as new cultural adaptations of some interventions (e.g., CLS-FUERTE in Mexico) enabled a cumulative and up-to-date presentation of the effectiveness of school-based ADHD interventions, even though the novel studies (i.e., 2018 onwards) couldn’t be statistically compared to the previous dataset due to the small sample size. Finally, considering the emergence of ADHD symptoms as early as age 4 and their strong link to the symptoms later in life (Lahey et al., 2016), for which it is important to design and evaluate early interventions to mitigate later academic and behavioural difficulties as shown in our subgroup analysis based on school type, we defined the age range of school-aged children to allow for a comprehensive understanding of ADHD interventions across developmental stages.

There were also some caveats. For example, we detected high heterogeneity in the meta-analyses of included studies across all outcomes. As discussed previously, this is a major problem and is prevalent in the meta-analysis of interventions that target such neurodiverse subgroups of the population, specifically autism and ADHD (Deniz et al., 2022, 2024; Francis et al., 2022; DuPaul et al., 2012). This may indicate that the included studies may not share a common effect size for the reported outcomes. Additionally, there was a considerable variation in intervention dosage, ranging from as little as 25 minutes to over 360 hours. Therefore, it was not possible to categorise interventions under few subgroups, in terms of their dosages, as any cut-off point or categorisation would have been arbitrary, and suffer from measurement error. Future reviews are strongly encouraged to investigate this matter, where possible. Moreover, our approach to choosing measurements for the meta-analyses aimed at continuity with a prior systematic review (Moore et al., 2018), as well as to increase validity of each studied domain, which meant that we included measurements in the same domains that Moore et al. (2018) reported. However, we recognise that the scopes of these scales were wide, including clinical performance measures, behavioural rating scales, and observational measures, which are different in terms of which aspect of the construct they measure. We therefore acknowledge that the included rating scales capture related constructs (e.g., core symptoms of ADHD) but different underlying mechanisms of these constructs (e.g., cognitive and behavioural aspects of ADHD). Combined with high heterogeneity, these findings should be interpreted cautiously. Another limitation is the lack of control for children who are prescribed a medicine for ADHD, as the majority of studies did not report this information. Nevertheless, by applying only between-group differences in this study, we aimed at omitting any positive/negative effects created by the medication use. Additionally, our approach to taking the first-reported outcome per rater in each target domain per study, although is a common practice in the field to reduce selective reporting bias (Deniz et al., 2022, 2024; Francis et al., 2022) does not rule out the possibility that applying a different data extraction strategy could provide different findings. Finally, unblinding and the lack of information on this matter in some of the included studies may mean that they may have promoted individuals’ masking behaviours instead of driving a meaningful change in core ADHD symptoms.

## Conclusion and implications

Overall, we found that school-based interventions are effective in reducing symptoms of ADHD, potentially only inattention, and externalising problems, and in improving academic and social skills. We found no evidence that such interventions also improve hyperactivity/impulsivity. Based on this, we suggest that school-based interventions are designed in a way that they target individuals’ attention deficits more than their hyperactivity/impulsivity. It is not clear, from our findings, why children with hyperactivity/impulsivity did not respond to such school-based interventions. We suggest, for the first time, such interventions should be more inclusive to target children who mainly have hyperactivity/impulsivity difficulties. Additionally, stronger effect sizes found in primary school settings, compared to secondary schools for inattention and academic outcomes further highlighted the importance of early intervention strategies, for children with ADHD. High heterogeneity remains to be an important issue, hence, more consistent practices and clear guidelines are needed to understand what intervention works, for whom, and in what dosage. Our review also showed that schools and teachers might benefit from interventions that directly build students’ cognitive, behavioural, and social skills through structured routines, explicit instructions, and consistent reinforcement.

## Data Availability

The original contributions presented in the study are included in the article/Supplementary material, further inquiries can be directed to the corresponding authors.
