# Supplementary material for: School-based randomized controlled trials for ADHD and accompanying impairments: a systematic review and meta-analysis
Source: Front Psychol. 2025 Jul 21;16:1611145. doi: 10.3389/fpsyg.2025.1611145 (PMC12318974; doi:10.3389/fpsyg.2025.1611145)
Supplement: Supplementary file 1 [file Supplementary_file_1.pdf]

Supplementary Materials - Figures

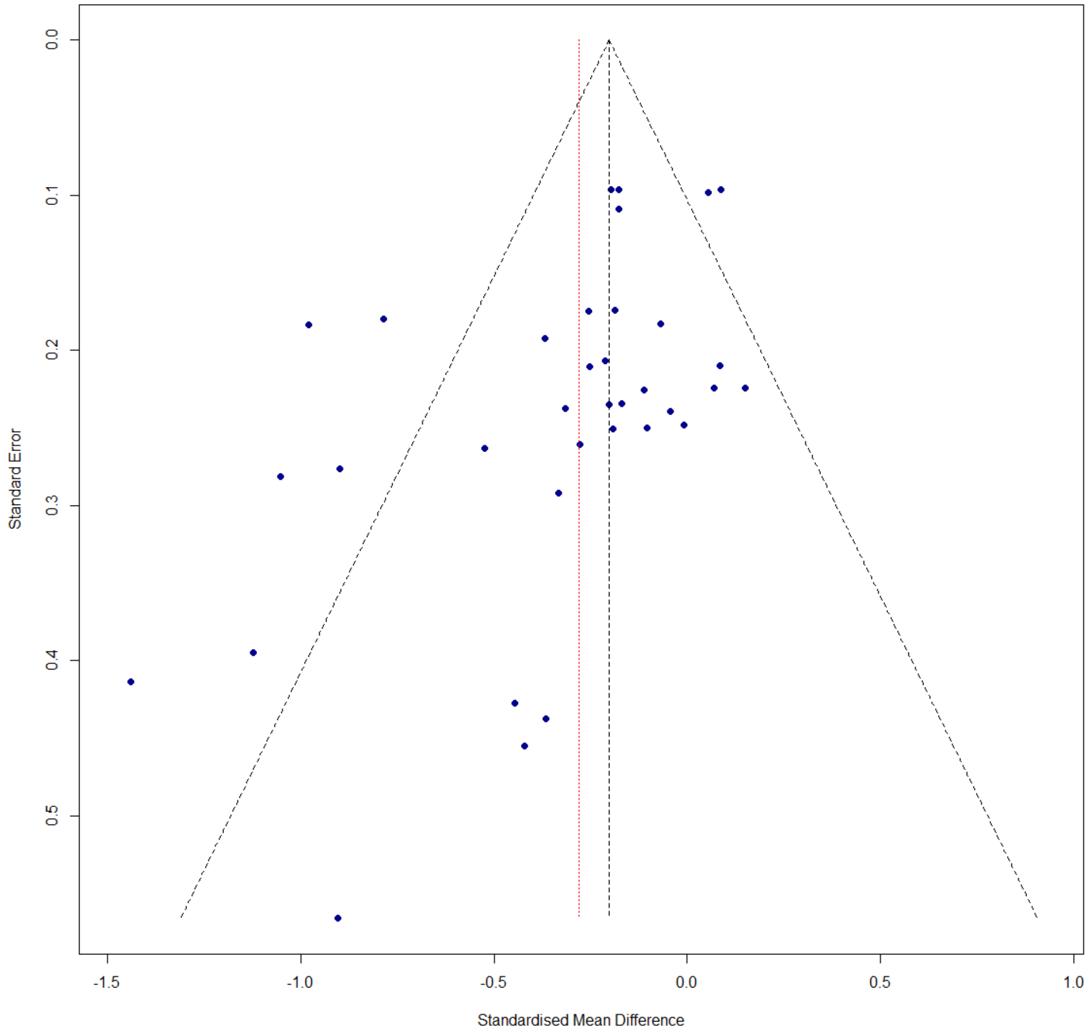

Figure S2. *Funnel Plot Showing Publication Bias in ADHD*

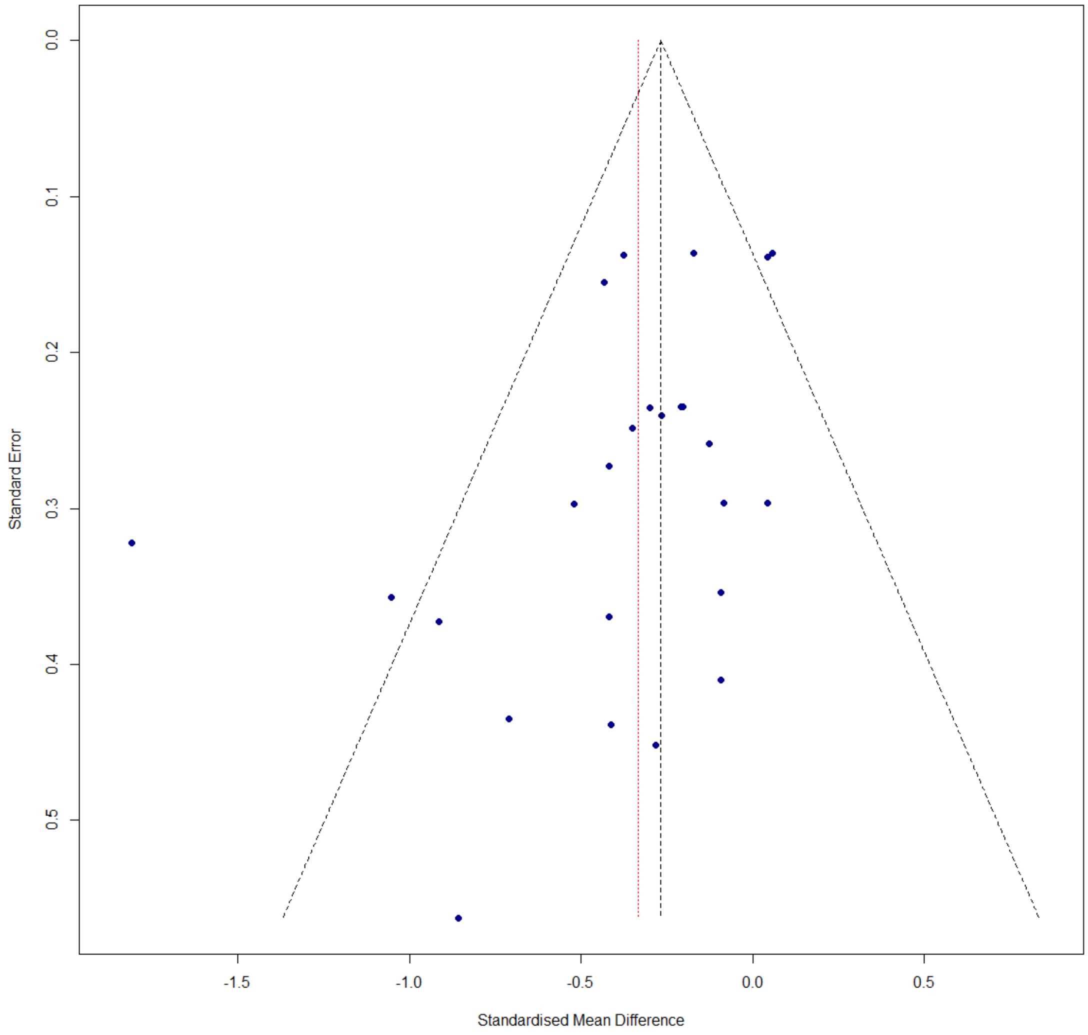

Figure S3. *Funnel Plot Showing Publication Bias in Inattention*

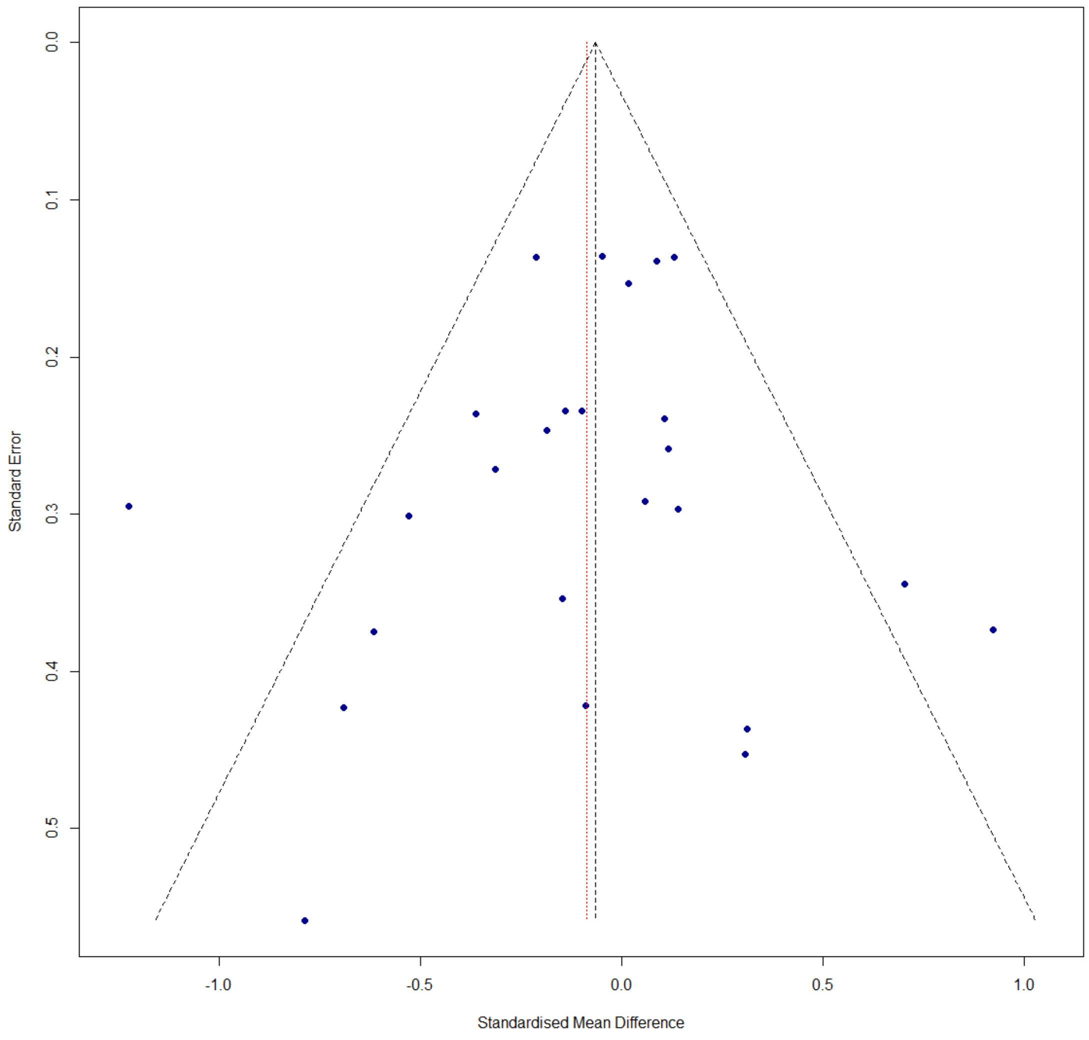

Figure S4. *Funnel Plot Showing Publication Bias in Hyperactivity*

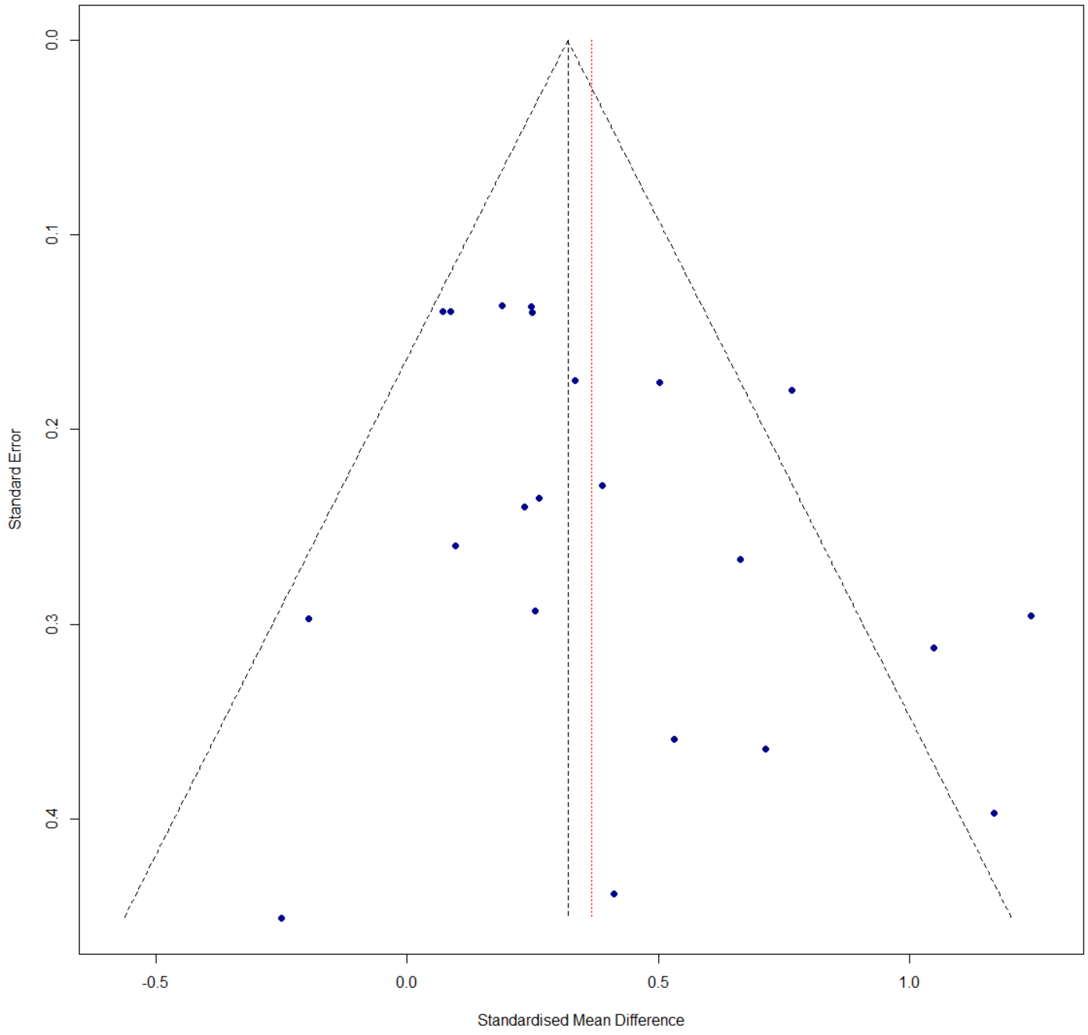

Figure S5. *Funnel Plot Showing Publication Bias in ADHD-related Impairment - Academic Skills*

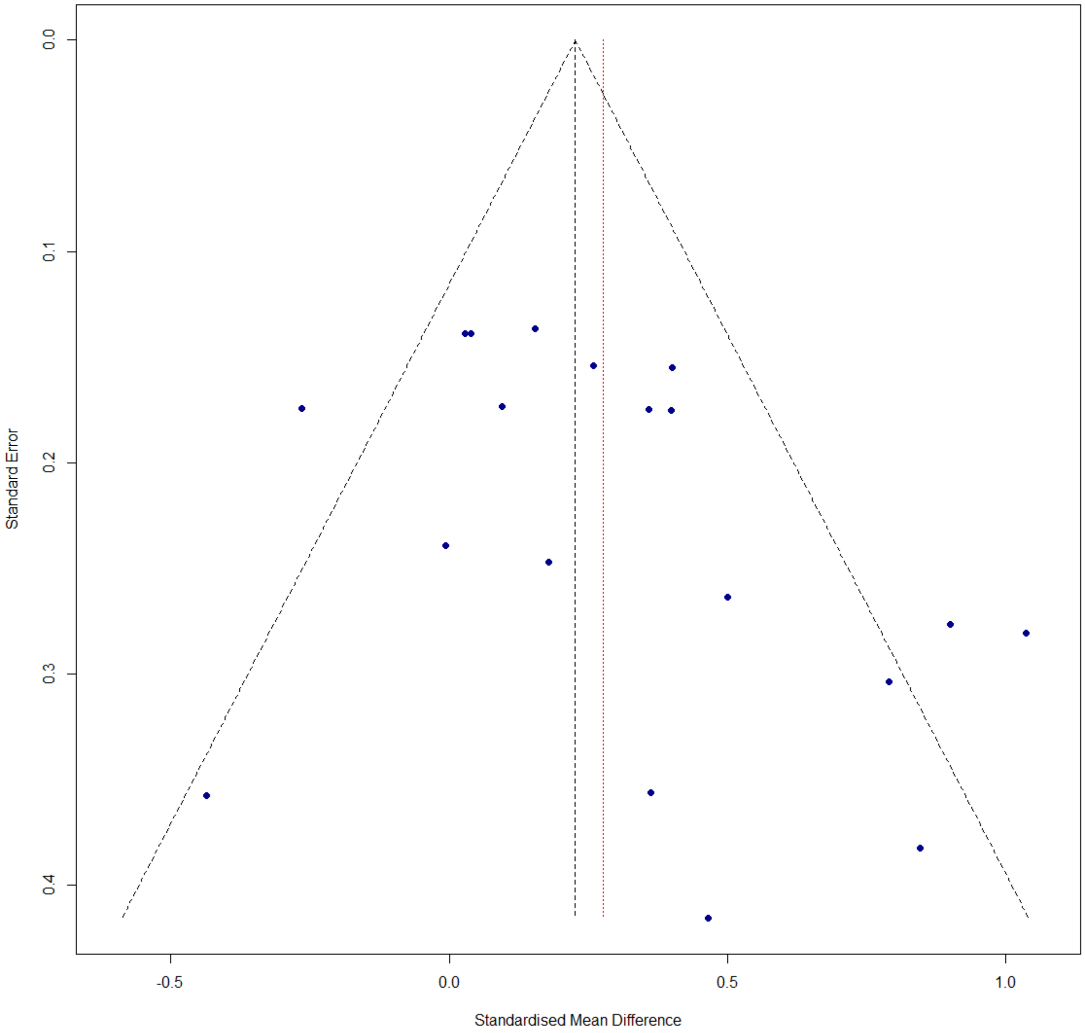

Figure S6. *Funnel Plot Showing Publication Bias in ADHD-related Impairment - Social Skills*

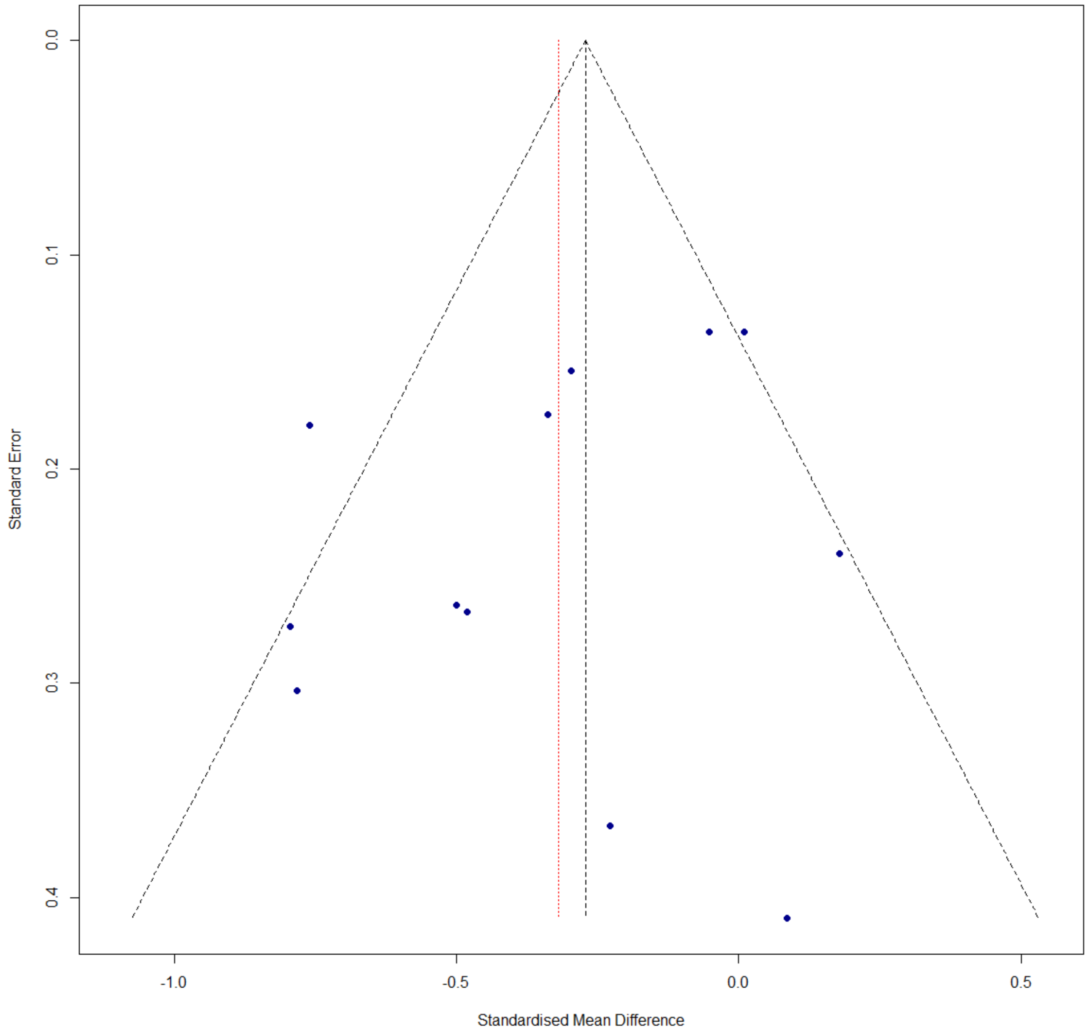

Figure S7. *Funnel Plot Showing Publication Bias in ADHD-related Impairment - Externalising Problems*
